# Semi-automatic detection of anteriorly displaced temporomandibular joint discs in magnetic resonance images using machine learning

**DOI:** 10.1186/s12903-025-06981-5

**Published:** 2025-10-10

**Authors:** Bin Ji, Yang Liu, Bin Zhou, Rui Mi, Yumeng Liu, Yungang Lv, Panying Wang, Yanjiao Li, Qingjun Sun, Nashan Wu, Yuping Quan, Songxiong Wu, Long Yan

**Affiliations:** 1 Radiology Department, Shenzhen University General Hospital, Shenzhen University Clinical Medical Academy, Shenzhen, 518055 People’s Republic of China; 2 Radiology Department, Shenzhen Qianhai Taikang Hospital, Shenzhen, 518055 People’s Republic of China

**Keywords:** Temporomandibular joint disc, Temporomandibular joint disorders, Machine learning, Magnetic resonance imaging

## Abstract

**Background:**

Accurate diagnosis of anterior disc displacement (ADD) is essential for managing temporomandibular joint disorders (TMJ). This study employed machine learning (ML) to automatically detect anteriorly displaced TMJ discs in magnetic resonance images (MRI).

**Methods:**

This retrospective study included patients with TMJ disorders who visited the Hospital between January 2023 and June 2024. Five machine learning models—decision tree (DT), K-nearest neighbors (KNN), support vector machine (SVM), random forest (RF), and logistic regression (LR)—were utilized to train and validate radiomics data derived from TMJ imaging. Model performance was assessed using an 8:2 train-test split, evaluating accuracy with metrics such as area under the curve (AUC), sensitivity, specificity, precision, and F1 score. After manual delineation of TMJ ROIs by an experienced radiologist (serving as reference standard), radiomic feature extraction included first-order statistics, size- and shape-based features, and texture features.The open-phase, close-phase, and open and close fusion radiomics image features were evaluated separately.

**Results:**

The study analyzed 382 TMJs from 191 patients, comprising 214 normal joints and 168 abnormal joints. The fusion radiomics model using five classifiers surpassed both open-phase and close-phase models, demonstrating superior performance in both training and validation cohorts. The fusion radiomics model consistently outperformed single-phase analyses across both diagnostic tasks. For normal vs. abnormal TMJ discrimination, the Random Forest (RF) classifier demonstrated robust performance with AUCs of 0.889 (95% CI: 0.854–0.924) in training and 0.874 (95% CI: 0.799–0.948) in validation.Complete performance metrics for all five classifiers are detailed in the main text.

**Conclusions:**

The fusion radiomics model effectively distinguished normal from abnormal joints and differentiated between ADDwR and ADDwoR, supporting personalized treatment planning.

**Clinical trial number:**

not applicable.

**Supplementary Information:**

The online version contains supplementary material available at 10.1186/s12903-025-06981-5.

## Background

 Temporomandibular joint disorders (TMJDs) encompass a spectrum of conditions characterized by anatomical, histological, and/or functional abnormalities involving the muscular and/or articular components of the temporomandibular joint (TMJ). These disorders are commonly associated with symptoms such as TMJ pain, restricted mandibular movement, and audible clicking or popping sounds during joint activity [1–3].With an estimated prevalence of approximately 31% in the general population [3], TMJDs exert a significant impact on patients’ quality of life, often contributing to psychological distress and imposing substantial healthcare costs [4, 5].Among these disorders, temporomandibular joint disc displacement (TMJDD) is the most prevalent, characterized by an abnormal relationship between the articular disc, the mandibular condyle, and the mandibular fossa [6]. The most frequently observed form of disc displacement, particularly in asymptomatic individuals, is anterior disc displacement (ADD), wherein the disc shifts anteriorly during mouth opening but relocates onto the condylar head during closure [7]. Two clinically significant subtypes of ADD have been identified: anterior disc displacement with reduction (ADDwR), where the disc returns to its normal position during jaw closure, and anterior disc displacement without reduction (ADDwoR), which involves persistent anterior displacement. ADDwoR is associated with chronic symptoms, including pain and joint dysfunction, and may contribute to progressive joint degeneration if not diagnosed and managed promptly [8].

Magnetic resonance imaging (MRI) is widely regarded as the gold standard for evaluating the soft tissue components of the TMJ, offering unparalleled resolution for visualizing fine anatomical structures [9].Its three-dimensional imaging capabilities enable dynamic assessment of the joint in motion, providing comprehensive visualization of disc position in both closed and open-mouth states [1].MRI is particularly useful for detecting variations in disc morphology and positional abnormalities, which are frequently associated with temporomandibular disorders (TMDs). ADD progresses through distinct stages, each with specific clinical and therapeutic implications [7]. However, due to the intricate anatomy of the TMJ, the small size of the articular disc, and potential variability in MRI image quality, interpreting these scans can be challenging, even for experienced clinicians [10, 11].

In recent years, machine learning (ML) has emerged as a transformative tool in medical imaging, enhancing the accuracy and efficiency of disease diagnosis and management [12–14]. When integrated with artificial intelligence (AI) techniques, ML offers significant advantages in processing large-scale medical datasets, enabling early detection and precise diagnostic capabilities. For example, convolutional neural networks (CNNs) [15], a subset of ML models, can be trained to differentiate between normal and pathological TMJ discs and to assess the extent of disc displacement, underscoring the potential of AI in medical image analysis.

Recent advances in deep learning (DL) have further expanded the diagnostic tools for TMJ disc displacement.The application of deep learning approaches related to different TMJ diseases and diagnostic problems was studied in [15].While these DL methods excel in capturing complex image patterns through end-to-end learning, radiomics offers distinct advantages in quantifying explicit and biologically interpretable features, such as texture heterogeneity and morphological dynamics across different jaw positions. Thus, the present study positions radiomics as a complementary approach to DL, particularly in leveraging multi-phase kinematic MRI to uncover biomechanical biomarkers of disc-condyle complex instability.

Beyond improving diagnostic accuracy, ML reduces the time required for image interpretation, facilitating faster and more precise clinical decision-making. This is particularly relevant for TMJDs such as ADD, where rapid and accurate diagnosis is critical for optimizing treatment outcomes and enhancing patients’ quality of life [16].Despite promising advances, current ML models face limitations in reliably distinguishing between ADDwR and ADDwoR, a critical differentiation in clinical practice that remains challenging.This study employed ML to automatically detect anteriorly displaced temporomandibular joint discs in MRI.Critically, for dynamic TMJ analysis requiring comparison of open- and closed-mouth states, radiomics enables direct extraction and quantification of biomechanical/morphological feature changes. This provides targeted insights into disc displacement mechanisms that end-to-end DL models may learn only implicitly through latent representations.

We hypothesize that the integration of radiomics features from multiple MRI phases will yield improved accuracy in distinguishing between normal and abnormal joints, as well as in differentiating between ADDwR and ADDwoR, when compared to analyses conducted using single-phase MRI data.

Our contributions were summarized as follows:

- Systematically analyze and compare the diagnostic value of different MRI phases to distinguish normal and abnormal joints or differentiate ADDwR and ADDwoR.
- Develop a fusion-phase radiomics model that combines features from open and close phases.
- The findings of this study could potentially enhance the accuracy for distinguishing normal and abnormal jointsor differentiating ADDwR and ADDwoR, and contribute to more personalized treatment strategies for distinguishing normal and abnormal jointsor differentiating ADDwR and ADDwoR.
- The clinical applicability of this integrated approach was validated with multiple classifiers.

## Methods

### Study design, patients, and data management

**Fig. 1:**
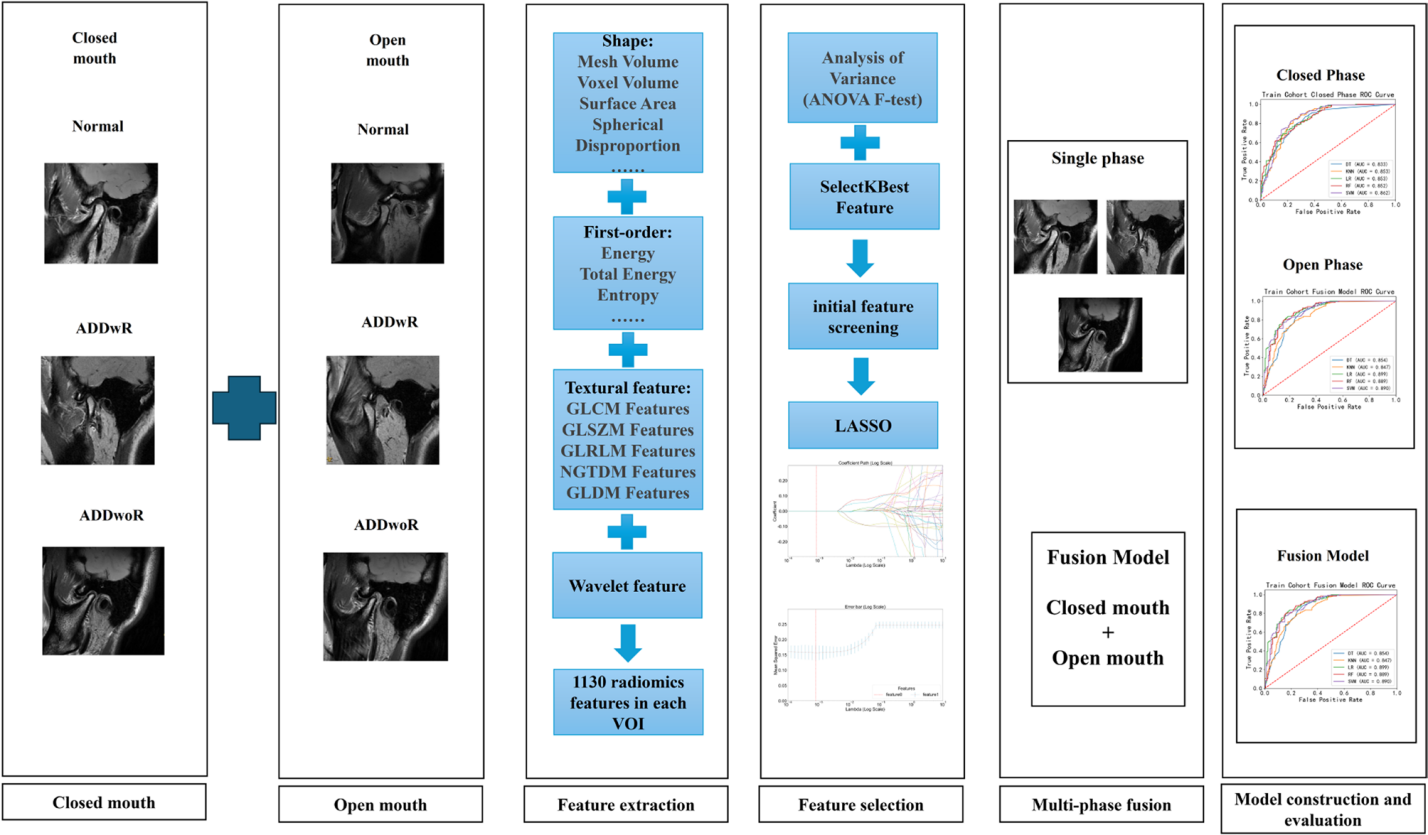
Flowchart of radiomics analysis

This retrospective study included patients who visited the Department of Radiology at Shenzhen University General Hospital for TMJDs between January 2023 and June 2024. This study was approved by the Ethical Committee of Shenzhen University General Hospital (Approval No.: KYLLMS-23). We confirm that all patient identifiers, including name, national ID number, birth date, examination date, accession numbers, institution name, and referring physician details, were permanently removed from the DICOM file headers and any associated clinical records prior to analysis. Only de-identified imaging data and the essential clinical labels required for the study (age, gender, and diagnosis) were retained. All subsequent analyses were performed exclusively using this anonymized dataset. This rigorous anonymization process was a core component of our approved protocol.The inclusion criteria were as follows: (1) clinical examination for suspected TMDs conducted by the same clinician in accordance with the “Diagnostic Criteria for Temporomandibular Disorders for Clinical and Research Applications” [17], (2) bilateral TMJ MRI performed, and (3) diagnosis of normal disc positioning (no displacement) or ADD in either or both TMJs. The exclusion criteria were as follows: (1) a history of hematological diseases, neurological disorders, oral inflammation, connective tissue diseases, TMJ trauma, oral cancer, or craniomaxillofacial surgery; (2) MRI scans deemed unsuitable for diagnostic evaluation due to prior operations, benign tumors, suspected adhesions, severe MRI artifacts, or cases involving lateral or posterior disc displacements; or (3) unresolved assessment discrepancies, defined as instances where evaluations by the dentist and radiologist were inconsistent and no consensus could be reached. The entire workflow of radiomics analysis was illustrated in Fig. 1.

### Magnetic resonance imaging

**Fig. 2:**
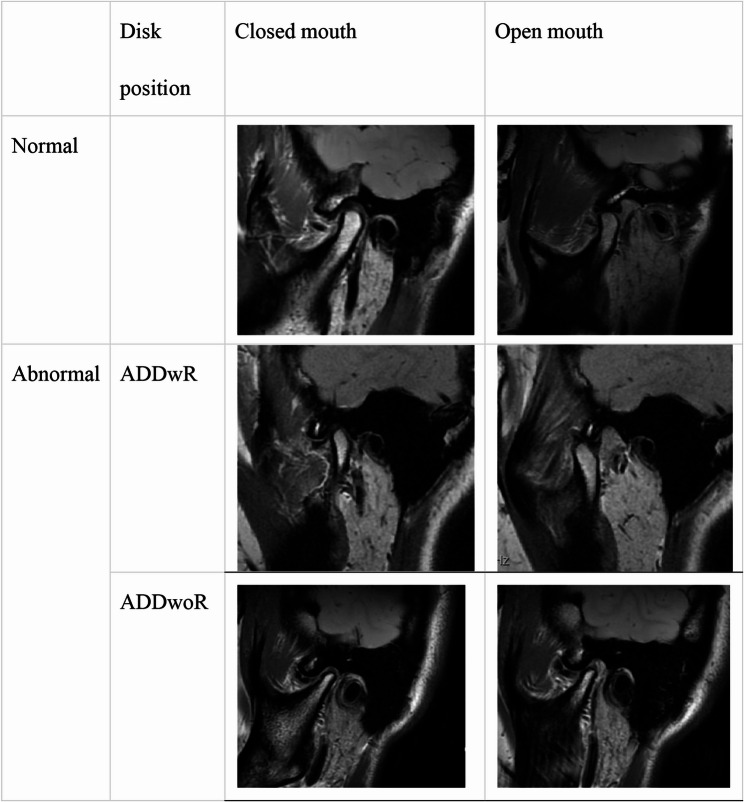
Magnetic resonance imaging (MRI) of normal and abnormal anterior disc locations with and without reduction. The posterior band of the joint disc is located at the top of the condyle. If the posterior band of the joint disc is located at 12 o’clock between the condyles, the joint disc position is considered normal. A disc located below 11 o’clock is considered anteriorly displaced. In the open oblique sagittal position, if the disco-condylar relationship returns to normal, it is reversible anterior disc displacement. If the condylar position of the articular disc cannot be restored in the open oblique sagittal position, it is irreversible anterior disc displacement

The TMJ MRI examinations were performed using a GE Discovery 750 3.0-T MRI system (General Electric, Milwaukee, WI, USA) equipped with an 8-channel SENSE head coil. Imaging was conducted in the axial, sagittal, and coronal planes using fast spin-echo (FSE) phases, with both closed-mouth and open-mouth positions routinely obtained. For bilateral TMJ imaging using oblique coronal scans (T2-weighted imaging, T2WI), the positioning line was aligned parallel to the transverse axis of the condylar process. For imaging in the closed-mouth position, the positioning line was perpendicular to the transverse axis of the condylar process. In the open-mouth position, a plastic mouth opener was placed between the patient’s upper and lower anterior teeth to facilitate maximum comfortable opening. Detailed acquisition parameters are provided in Supplementary Material S1. Disc displacement on MRI was classified into three categories—normal, ADDwR, and ADDwoR—based on the criteria described by Orhan et al. [18]. A TMJ was considered normal when the posterior band of the articular disc was positioned at the 12 o’clock position relative to the superior contour of the condyle. Deviations from this position were classified as either ADDwR or ADDwoR. All MRI images were independently evaluated and interpreted twice by two radiologists with over 10 years of experience in TMJ imaging. In cases of disagreement between their assessments, consensus was reached through repeated evaluation and discussion. The diagnostic criteria used for image interpretation are detailed in Fig. 2.

### Region of interest segmentation

**Fig. 3:**
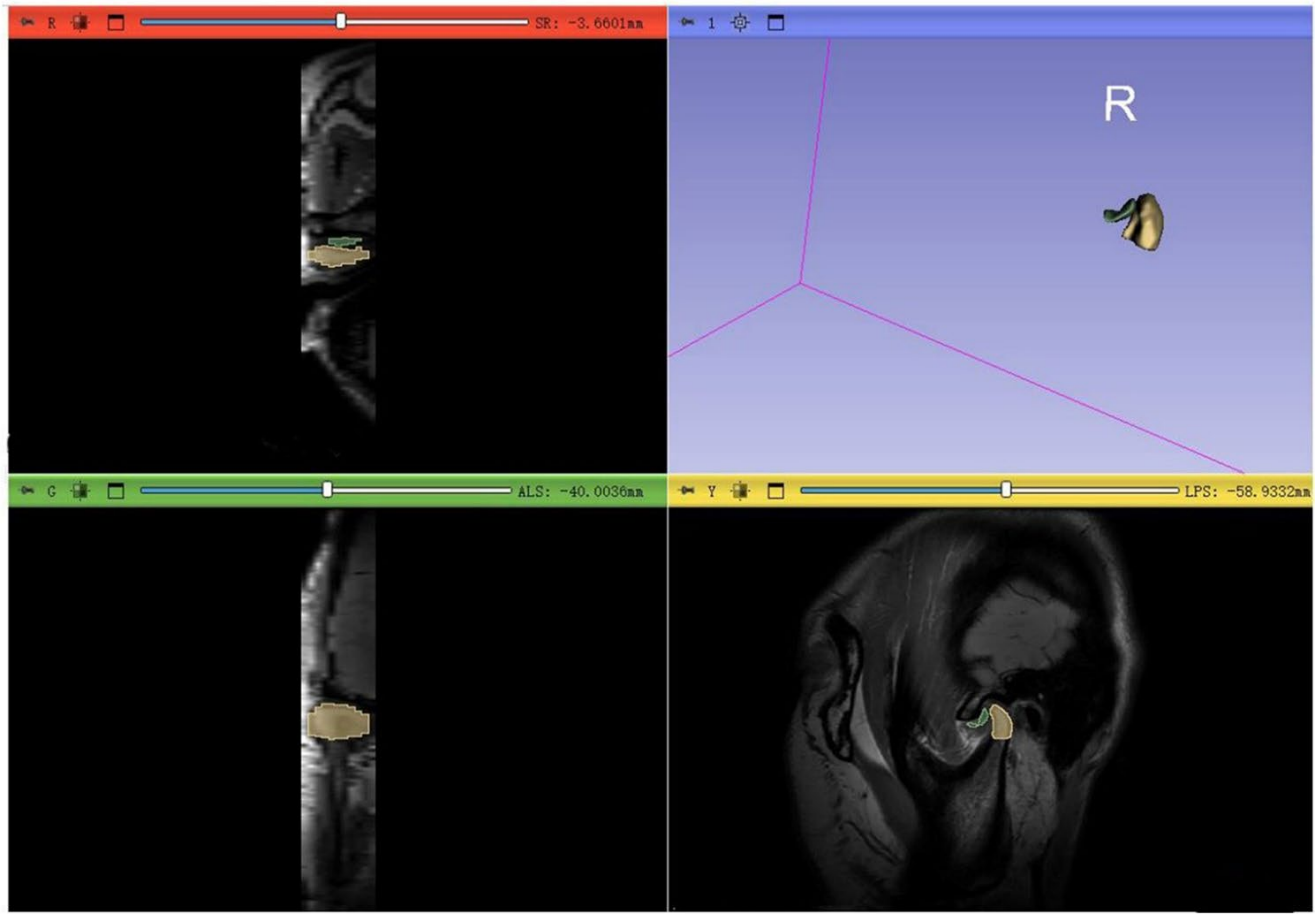
Magnetic resonance imaging (MRI) images showing the tracings of the volume of interest (VOI) in a radiomic (3D-Slice) platform

To improve classification accuracy, the AI-assisted diagnostic process was divided into two stages, focusing on isolating the relevant anatomical structures and eliminating extraneous information (e.g., the skull or air) from the original MRI images. In the first stage, a region of interest (ROI) was manually delineated by an experienced board-certified radiologist to encompass the joint cavity on sagittal MRI images using the 3D Slicer software (University of South Carolina, https://www.slicer.org/). This step was performed by a single radiologist with over 10 years of experience.The diagnosis of disc displacement was predominantly contingent upon the morphological characteristics and signal attributes of the TMJ disc, as well as the spatial relationship between the disc and the condyle. In the context of this study, the ROI specifically concentrated on the structural analysis of the TMJ disc and condyle, as illustrated in the sagittal plane. The TMJ disc was manually delineated on each MRI slice (Fig. 3).This manual cropping process ensured accurate outlining of each ROI and maintained consistency in image positioning. By reducing the size of the images compared to the originals, this step enhanced the precision of the subsequent binary classification task. In the second stage, the cropped ROIs were categorized into three groups: normal, ADDwR, and ADDwoR. By focusing specifically on the disc and condyle, this two-stage approach reduced the influence of irrelevant anatomical structures, thereby improving the performance of the classification model.

To ensure feature reliability, an experienced board-certified radiologist repeated manual delineation of 30 randomly selected cases after two months. Intra-observer consistency was quantitatively evaluated using both the dice similarity coefficient (DSC) for spatial agreement and intraclass correlation coefficients (ICCs) for feature stability.

### Feature extraction and selection

PyRadiomics 3.0.1 (https://pyradiomics.readthedocs.io/en/latest/) was used to extract and analyze radiomic features. Image normalization was performed using a ratio of 50, and resampling was conducted with the B-Spline interpolator method based on the average spacing of the training dataset. The extracted radiomic features included first-order statistics, shape-based features (2D and 3D), and texture-based features, such as the gray level co-occurrence matrix (GLCM), gray level size zone matrix (GLSZM), gray level run length matrix (GLRLM), neighboring gray-tone difference matrix (NGTDM), and gray level dependence matrix (GLDM). All features were extracted using the default PyRadiomics settings. Additionally, features were derived from Laplacian of Gaussian (LoG) filtering (with sigma values of 1.0, 3.0, and 5.0) and wavelet decomposition (using the Coiflet 1 wavelet function). Detailed descriptions of these features are available in the PyRadiomics documentation (https://pyradiomics.readthedocs.io/en/latest/). A total of 1,130 radiomic features were extracted from the MRI data of patients diagnosed with normal, ADDwR, and ADDwoR.

Radiological features of the normal group, ADDwR group, and ADDwoR group were selected through the following two steps, involving open-phase, close-phase, and fusion radiomics: (1) The top 50 features most relevant to the classification of ADD were identified by calculating the MI between each feature and the classification labels. (2) These top features were further refined using the least absolute shrinkage and selection operator (LASSO), which eliminated redundant or irrelevant features and retained only the most representative ones. This process resulted in the final set of radiomic features used for subsequent analysis.

### Model development and validation

**Fig. 4:**
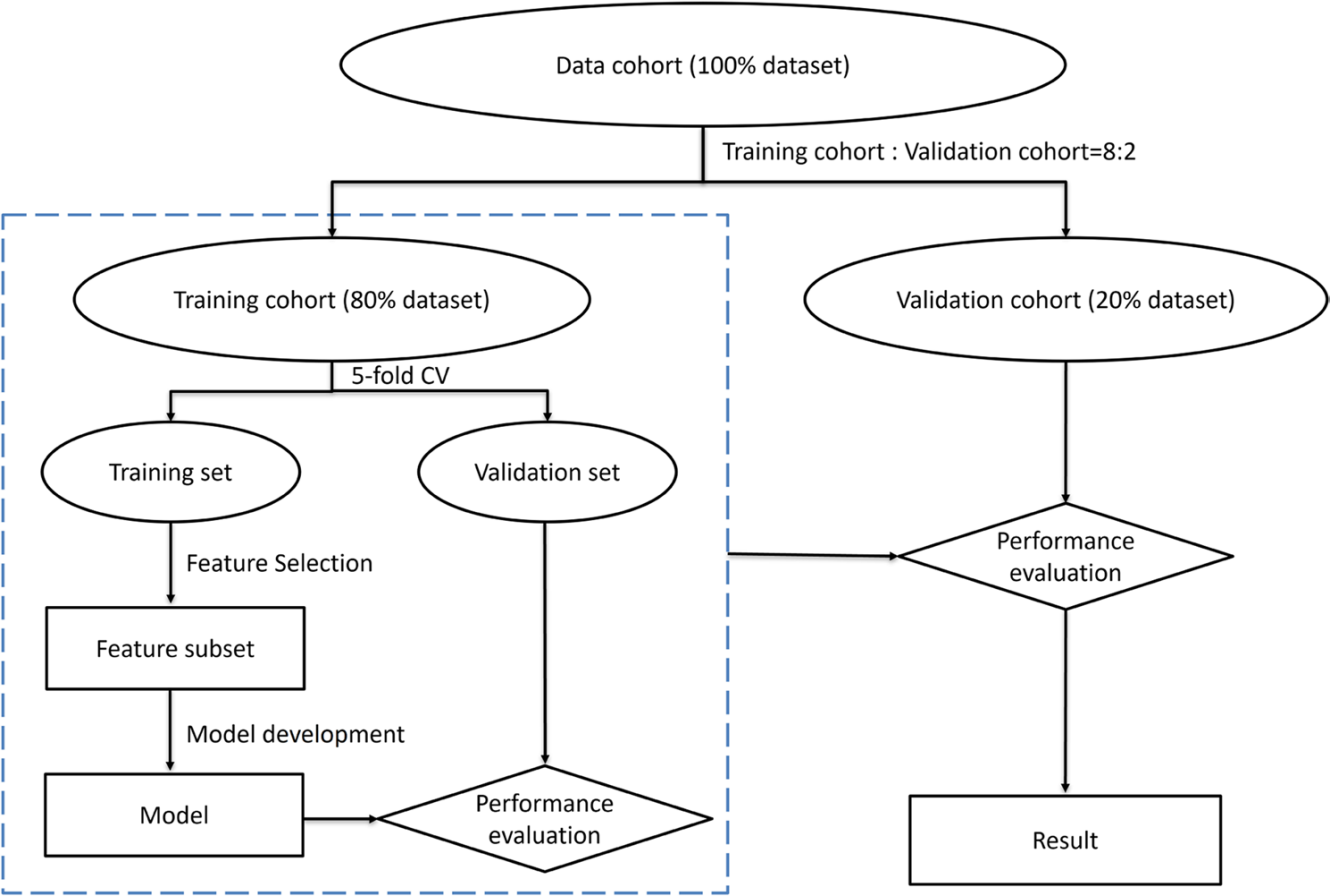
The Entire Process of Model Analysis

Five classifiers, including decision tree (DT), K-nearest neighbors (KNN), logistic regression (LR), random forest (RF), and support vector machine (SVM), were used to develop diagnostic models for ADD pathologies. The study population was randomly divided into a training cohort and a validation cohort in an 8:2 ratio. The model parameters were then optimized in the training cohort using a five-fold cross-validation scheme. In the cross-validation process, the four-fold data were used as the training dataset, and the one-fold data were used as the validation dataset, which was repeated five times, and then the predicted probability of the five-fold validation data was used as a whole to evaluate the performance of the model. During the independent validation cohort, we evaluated model performance with the predicted probabilities of models produced by cross-validation. The entire workflow of model analysis was illustrated in Fig. 4.

### SHAP analysis

After building the model, we performed SHAP analysis to interpret how individual features contribute to their predictions. We computed SHAP values for each feature. To illustrate feature importance and their impact on classifying both normal and abnormal using an LR classifierin the Fusion Model, we created visualizations including the SHAP Feature Importance plot and the SHAP Summary plot.

### Statistical analysis

All statistical analyses were performed using MedCalc version 20.0.19.7 (2011 MedCalc Software Bvba, Mariakerke, Belgium), SPSS version 20.0.0 (IBM, Armonk, NY, USA), and Python 3.6.12 (https://www.python.org). The area under the curve (AUC) values for the models were compared using the DeLong method. All statistical tests were two-sided, with a significance level set at *P* < 0.05. The AUC was utilized to evaluate the discriminatory ability of the models. Sensitivity, specificity, F1-score, and precision were calculated based on the optimal cut-off value, which was determined by maximizing the Youden index.

## Results

### Subjective evaluation

This study analyzed the TMJ characteristics of 191 patients, of whom157 (82.2%) were female and 34 (17.8%) were male. The mean age of the patients was 26.96 ± 6.74 years. A total of 382 TMJs were included in the analysis, with 214 (56.4%) classified as normal and 168 (43.6%) classified as abnormal. Among the 168 TMJs diagnosed with abnormalities, the most common type was ADDwoR, accounting for 111/168 (66.1%), while ADDwR comprised 57/168 (33.9%)(Table 1).

**Table 1:** Characteristics of the patients according to their TMJ

Variable	All (*n* = 191)	Normal TMJ (*n* = 107)	Abnormal TMJ (*n* = 168)
Age (years)	26.96 ± 6.74	27.54 ± 6.57	26.32 ± 6.90
Sex			
Male	34 (17.8%)	21 (19.6%)	12 (14.0%)
Female	157 (82.2%)	86 (80.4%)	72 (86.0%)
Number of TMJs	382	214 (56.0%)	168 (44.0%)
ADDwR	57	-	57 (33.9%)
ADDwoR	111	-	111 (66.1%)

*TMJ* temporomandibular joint, *ADDwR* anterior disc displacement with reduction, *ADDwoR* anterior disc displacement without reduction

Spatial agreement between the original and repeated segmentations, assessed using the Dice Similarity Coefficient (DSC), demonstrated excellent reproducibility (mean DSC = 0.988 ± 0.011). Feature stability across the repeated delineations was evaluated via Intraclass Correlation Coefficients (ICCs) for all 1130 extracted features, all yielding valid results. The overall feature reliability was exceptionally high, with amean ICC of 0.989 ± 0.050. Classification based on established ICC agreement thresholds revealed that the vast majority of features (98.1%) exhibited excellent agreement (ICC ≥ 0.8), while1.1%showed moderate agreement (ICC 0.5–0.79), and0.8%showed poor agreement (ICC < 0.5), confirming that higher ICC values correspond to greater consistency between the physician’s repeated delineations.

### Prediction results of the five classifiers for normal TMJ vs. ADD

**Fig. 5:**
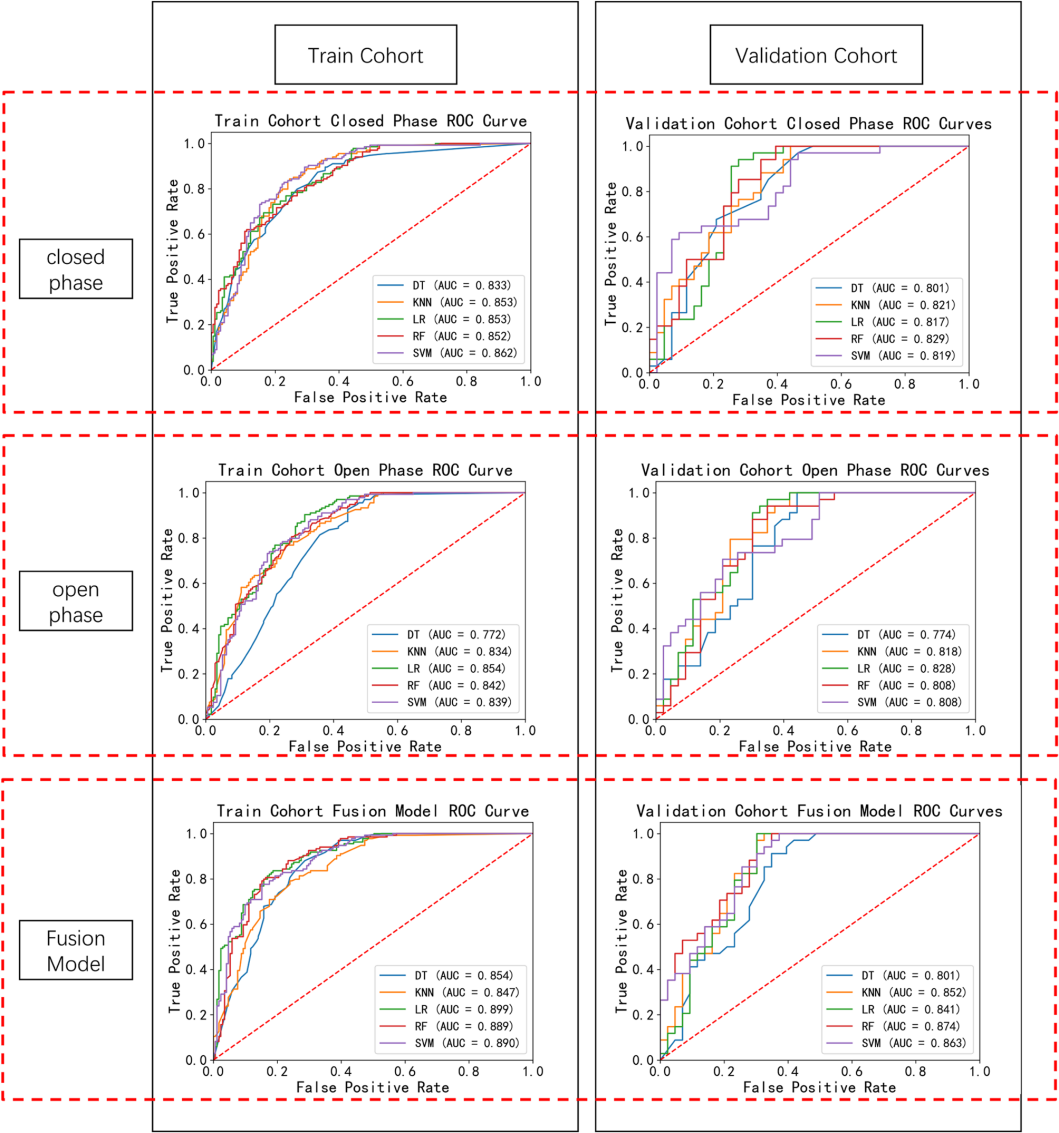
Receiver operating characteristic (ROC) curves for normal and abnormal classifications using five classifiers in the training cohort (*n* = 305) and validation cohort (*n* = 77) across three experimental conditions: closed phase, open phase, and fusion model. DT: decision tree; KNN: K-nearest neighbors; LR: logistic regression; RF: random forest; SVM: support vector machine; AUC: area under the curve

The diagnostic performance of the KNN, DT, LR, SVM, and RF models for distinguishing normal from abnormal TMJs was illustrated in the three models(open-phase, close-phase, and fusion models)(Table 2 and Fig. 5). The fusion radiomics model, using five classifiers, outperformed the open-phase and close-phase models, demonstrating superior performance in both the training cohort and validation cohort.The results for the five classifiers were DT (95% CI:0.814–0.894, AUC = 0.854, F1 = 0.784), KNN (95% CI:0.807–0.887, AUC = 0.847, F1 = 0.754),SVM (95% CI:0.855–0.925,AUC = 0.890, F1 = 0.788), LR (95% CI:0.865–0.933,AUC = 0.899, F1 = 0.806), and RF (95% CI:0.854–0.924,AUC = 0.889, F1 = 0.788) in the training cohort, and 0.801 (95% CI: 0.712–0.890), 0.852 (95% CI: 0.773–0.931),0.863 (95% CI: 0.786–0.939), 0.841 (95% CI: 0.759–0.923), and 0.874 (95% CI: 0.799–0.948) in the validation cohort, respectively.

**Table 2:** Assessment of diagnostic performance using five classifiers for normal and abnormal classifications in closed phase, open phase, and fusion model across both training and validation cohorts

	Classifier	Training cohort(*n* = 305)						Validation cohort(*n* = 77)					
		AUC	F1	Sen	Spe	Pre	*P*	AUC	F1	Sen	Spe	Pre	*P*
closed phase	DT	0.772	0.743	0.925	0.556	0.620	*	0.774	0.782	1.000	0.558	0.642	*
	KNN	0.834	0.736	0.769	0.749	0.705	*	0.818	0.791	1.000	0.581	0.654	*
	LR	0.854	0.786	0.903	0.690	0.695	*	0.828	0.805	0.971	0.651	0.688	*
	RF	0.842	0.750	0.806	0.731	0.701	*	0.808	0.790	0.941	0.651	0.681	*
	SVM	0.839	0.766	0.881	0.673	0.678	*	0.808	0.716	0.706	0.791	0.727	*
open phase	DT	0.833	0.760	0.873	0.667	0.672	*	0.801	0.759	0.971	0.535	0.623	*
	KNN	0.853	0.782	0.843	0.754	0.729	*	0.821	0.782	1.000	0.558	0.642	*
	LR	0.853	0.74	0.731	0.807	0.748	*	0.817	0.821	0.941	0.721	0.727	*
	RF	0.852	0.741	0.791	0.731	0.697	*	0.829	0.800	1.000	0.605	0.667	*
	SVM	0.862	0.789	0.896	0.708	0.706	*	0.819	0.712	0.618	0.907	0.84	*
Fusion Model	DT	0.854	0.784	0.881	0.713	0.707	*	0.801	0.775	0.912	0.651	0.674	*
	KNN	0.847	0.754	0.791	0.760	0.721	*	0.852	0.829	1.000	0.674	0.708	*
	LR	0.899	0.806	0.836	0.813	0.778	*	0.841	0.84	1.000	0.698	0.723	*
	RF	0.889	0.808	0.881	0.766	0.747	*	0.874	0.819	1.000	0.651	0.694	*
	SVM	0.890	0.788	0.776	0.848	0.800	*	0.863	0.810	1.000	0.628	0.680	*

*SVM* support vector machine, *LR* logistic regression, *RF* random forest, *DT* decision tree, *KNN* K-nearest neighbors, *CI* confidence interval, *AUC* area under the receiver operating characteristic curve

^a^* P* value is the significance level for comparing the AUC with a random case (AUC=0.5), *, *p* < 0.0001

### Prediction results of the five classifiers for ADDwR vs. ADDwoR among patients with ADD

**Fig. 6:**
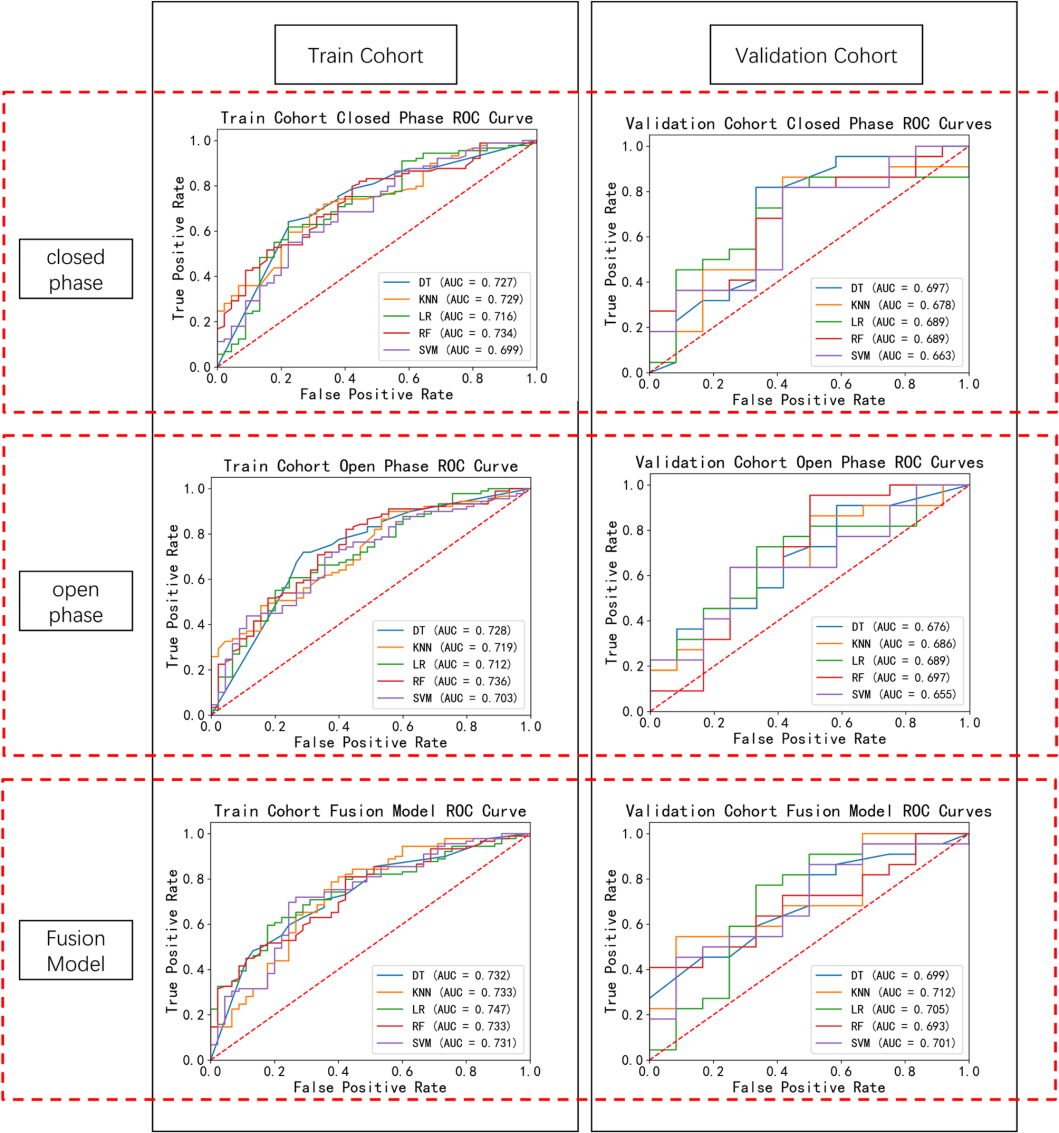
Receiver operating characteristic (ROC) curves for the ADDwR and ADDwoR classifications using five classifiers in the training cohort (*n* = 134) and validation cohort (*n* = 34) across three experimental conditions: closed phase, open phase, and fusion model. DT: decision tree; KNN: K-nearest neighbors; LR: logistic regression; RF: random forest; SVM: support vector machine; AUC: area under the curve

For diagnosing ADDwR versus ADDwoR, the diagnostic efficacy of the five classifiers was slightly reduced in three models (Table 3 and Fig. 6).The results for the five classifiers were DT (95% CI:0.657–0.807, AUC = 0.732, F1 = 0.806), KNN (95% CI:0.658–0.808,AUC = 0.733, F1 = 0.804),SVM (95% CI:0.656–0.806,AUC = 0.731, F1 = 0.776), LR (95% CI:0.673–0.821,AUC = 0.747, F1 = 0.707), and RF (95% CI:0.658–0.808,AUC = 0.733, F1 = 0.8) in the training cohort, and 0.699 (95% CI: 0.545–0.853), 0.712 (95% CI: 0.559–0.864),0.701 (95% CI: 0.547–0.855), 0.705 (95% CI: 0.551–0.858), and 0.693 (95% CI: 0.537–0.848) in the validation cohort, respectively.

**Table 3:** Assessment of diagnostic performance using five classifiers for the ADDwR and addwor classifications in closed phase, open phase, and fusion model across both training and validation cohorts

	Classifier	Training cohort(*n* = 134)						Validation cohort(*n* = 34)					
		AUC	F1	Sen	Spe	Pre	*P*	AUC	F1	Sen	Spe	Pre	*P*
closed phase	DT	0.727	0.731	0.640	0.778	0.851	*	0.697	0.818	0.818	0.667	0.818	*
	KNN	0.729	0.762	0.719	0.667	0.81	*	0.678	0.826	0.864	0.583	0.792	*
	LR	0.716	0.714	0.618	0.778	0.846	*	0.689	0.800	0.818	0.583	0.783	*
	RF	0.734	0.802	0.820	0.556	0.785	*	0.689	0.800	0.818	0.583	0.783	*
	SVM	0.699	0.662	0.551	0.778	0.831	*	0.663	0.800	0.818	0.583	0.783	*
open phase	DT	0.728	0.771	0.719	0.711	0.831	*	0.676	0.816	0.909	0.417	0.741	*
	KNN	0.719	0.825	0.899	0.444	0.762	*	0.686	0.718	0.636	0.750	0.824	*
	LR	0.712	0.701	0.607	0.756	0.831	*	0.689	0.762	0.727	0.667	0.800	*
	RF	0.736	0.807	0.820	0.578	0.793	*	0.697	0.857	0.955	0.500	0.778	*
	SVM	0.703	0.753	0.719	0.622	0.79	*	0.655	0.718	0.636	0.750	0.824	*
Fusion Model	DT	0.732	0.806	0.843	0.511	0.773	*	0.699	0.783	0.818	0.500	0.750	*
	KNN	0.733	0.804	0.809	0.600	0.800	*	0.712	0.686	0.545	0.917	0.923	*
	LR	0.747	0.707	0.596	0.822	0.869	*	0.705	0.791	0.773	0.667	0.81	*
	RF	0.733	0.800	0.809	0.578	0.791	*	0.693	0.581	0.409	1.000	1.000	*
	SVM	0.731	0.776	0.719	0.733	0.842	*	0.701	0.606	0.455	0.917	0.909	*

*SVM* support vector machine, *LR* logistic regression, *RF* random forest, *DT* decision tree, *KNN* K-nearest neighbors, *CI* confidence interval, *AUC* area under the receiver operating characteristic curve

^a^*P* value is the significance level for comparing the AUC with a random case (AUC = 0.5)

### Lasso regression for feature selection and SHAP analysis

**Fig. 7:**
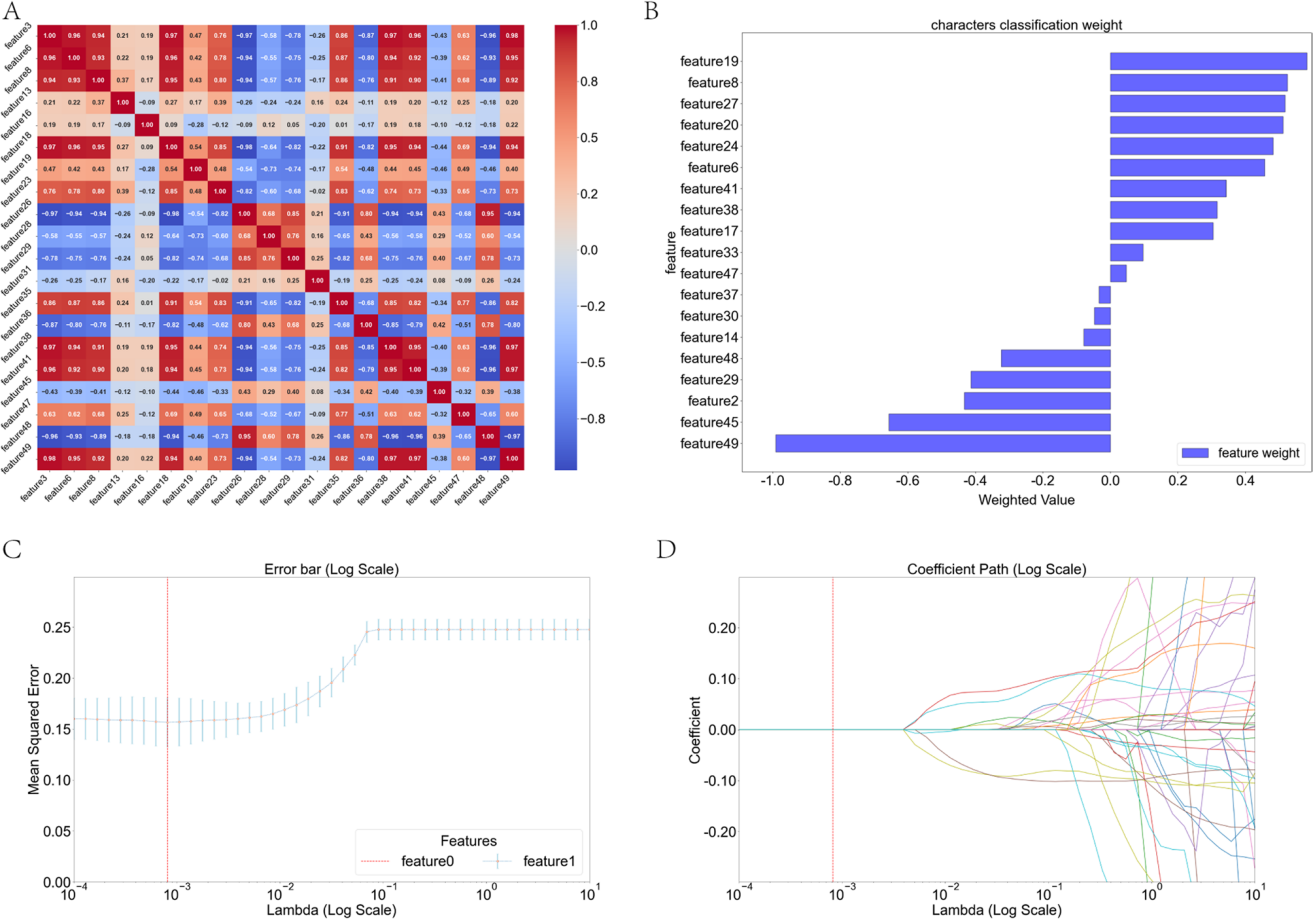
LASSO regression analysis for feature selection. **A** Feature correlation heatmap. **B** LASSO coefficient trajectories across lambda values. **C** Cross-validated mean squared error vs. lambda. **D** LASSO-selected feature coefficients with error bars

Our L1-regularized LASSO-CV framework demonstrated significant efficacy in radiomics feature optimization (Fig. 7A and D; Table 4). The feature correlation heatmap (Fig. 7A) revealed a *predominantly*weak-correlation structure (|*r*| < 0.3 in > 70% of matrix cells), ideal for stable modeling. Through coefficient shrinkage at the optimal λ = 0.0008 (Fig. 7D), 30 redundant features were compressed to zero, preserving 20 non-redundant predictors.The classification weight plot (Fig. 7B) confirmed multi-scale feature dominance: wavelet-HLH_glcm_Imc1_RO (feature19, + 0.526) exhibited the strongest positive weighting, whereas log-sigma-5-0-mm-3D_glrlm_GrayLevelNonUniformity_RC (feature49, − 0.996) was the top negative predictor. Retained features comprised original shape (23%), wavelet-transformed (60%), andlog-sigma filtered (17%) radiomics.Model stability was evidenced by the error-bar profile (Fig. 7C): MSE plateaued at 0.1566 ± 0.0098 for λ > 0.2, with coefficient paths converging robustly at λ = 10⁻³ (vertical red line, Fig. 7D). The 60% dimensionality reduction (50 → 20 features) did not compromise predictive power (cross-validation MSE fluctuation: ±6.3%).

**Table 4:** Double-Validated radiomics signature: LASSO coefficients and SHAP feature importance of selected features

Feature ID	Feature Name	Type	LASSO Coef	SHAP Value	Weight Direction
feature3	original_shape_Maximum2DDiameterSlice_RO	Original Shape	−0.305251	-	Negative
feature6	original_gldm_DependenceNonUniformity_RO	GLDM	+ 0.432238	+ 0.05	Dominant (+)⁺
feature8	wavelet-LLH_gldm_GrayLevelNonUniformity_RO	Wavelet-GLDM	+ 0.510495	-	Positive
feature13	wavelet-LHH_ngtdm_Busyness_RO	Wavelet-NGTDM	−0.076439	-	Negative
feature16	wavelet-HLL_glrlm_HighGrayLevelRunEmphasis_RO	Wavelet-GLRLM	+ 0.298745	-	Positive
feature18	wavelet-HLL_gldm_GrayLevelNonUniformity_RO	Wavelet-GLDM	+ 0.513720	+ 0.04	Dominant (+)⁺
feature19	wavelet-HLH_glcm_Imc1_RO	Wavelet-GLCM	+ 0.526569	+ 0.02	Positive
feature23	wavelet-HHL_glszm_LargeAreaEmphasis_RO	Wavelet-GLSZM	+ 0.509587	+ 0.02	Positive
feature26	wavelet-HHL_ngtdm_Coarseness_RO	Wavelet-NGTDM	+ 0.595405	+ 0.02	Positive
feature28	wavelet-HHH_glrlm_RunLengthNonUniformityNorm_RO	Wavelet-GLRLM	−0.408860	-	Negative
feature29	wavelet-HHH_gldm_DependenceEntropy_RO	Wavelet-GLDM	−0.047443	+ 0.03	Negative
feature31	wavelet-HHH_ngtdm_Busyness_RO	Wavelet-NGTDM	+ 0.099538	-	Positive
feature35	log-sigma-3-0-mm-3D_gldm_DependenceNonUniformity	Log-Sigma Filter	−0.017475	-	Negative
feature36	original_shape_Flatness_RC	Original Shape	+ 0.297256	-	Positive
feature38	original_shape_Maximum3DDiameter_RC	Original Shape	−0.105099	-	Negative
feature41	original_glrlm_GrayLevelNonUniformity_RC	GLRLM	+ 0.347918	+ 0.03	Positive
feature45	wavelet-HLL_glcm_ClusterShade_RC	Wavelet-GLCM	−0.659098	+ 0.04	Dual Role⁰
feature47	wavelet-LLL_glszm_LargeAreaHighGrayLevelEmphasis_RC	Wavelet-GLSZM	+ 0.049312	-	Positive
feature48	wavelet-LLL_ngtdm_Coarseness_RC	Wavelet-NGTDM	−0.338163	-	Negative
feature49	log-sigma-5-0-mm-3D_glrlm_GrayLevelNonUniformity_RC	Log-Sigma Filter	−0.995950	-	Domina

• Impact Direction classification: Dominant (+): LASSO coef > 0.5 or SHAP ≥ 0.04; Dominant (-): LASSO coef < −0.6

• Dual Role: Features exhibiting contradictory directional impactsbetween LASSO (embedded selection) and SHAP (post-hoc interpretation) frameworks, indicating context-dependent nonlinearity or subgroup-specific effects

**Fig. 8:**
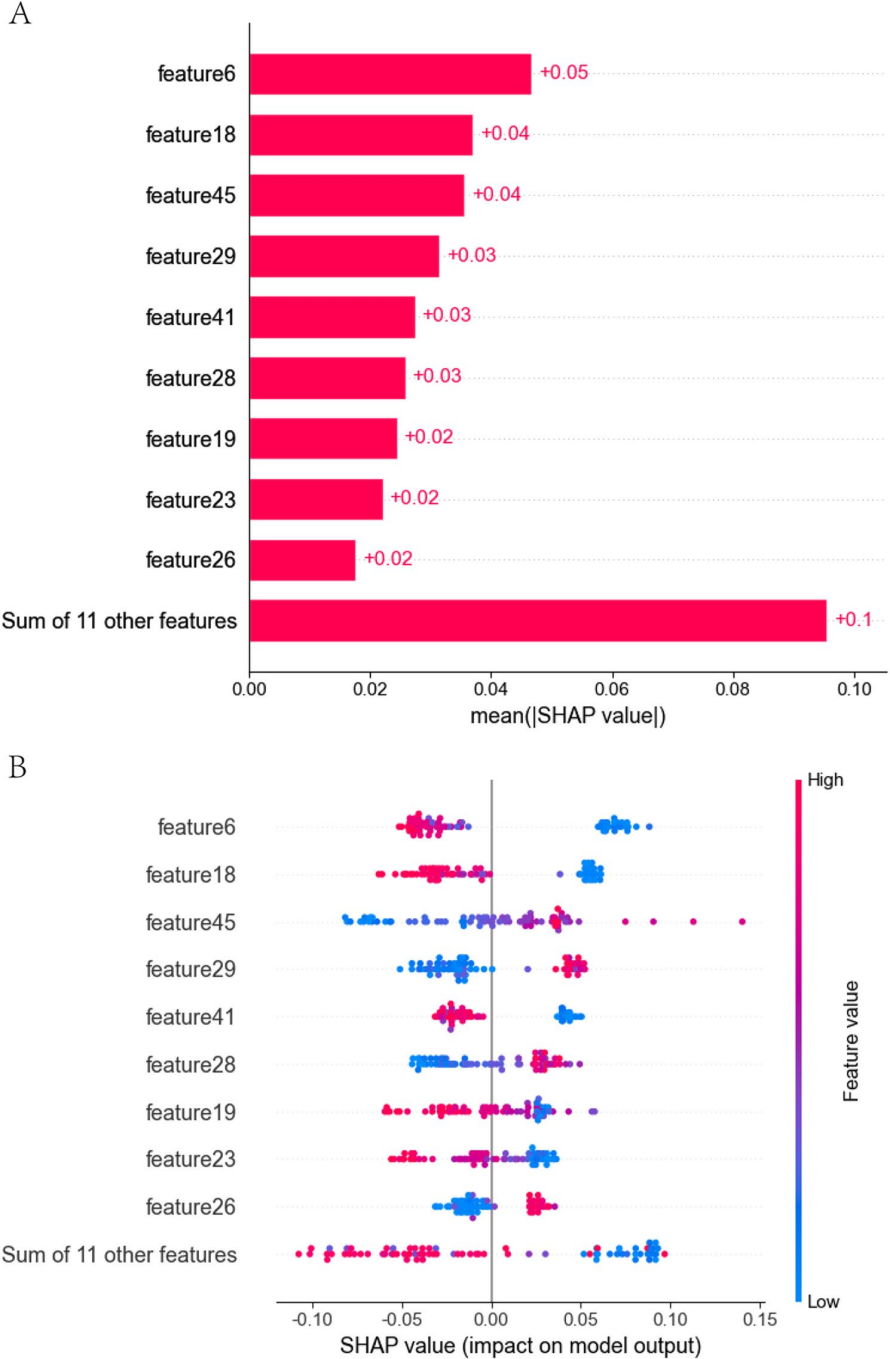
SHAP visualization of global feature importance (**A**) and SHAP global colony plot (**B**) by the fusion model using the LR model

SHAP analysis (Fig. 8A and B; Table 4) revealed nonlinear feature contributions to model outputs. The global importance plot (Fig. 8A) identified feature6 (original_gldm_DependenceNonUniformity_RO) as the top contributor (mean |SHAP| = +0.05), followed by wavelet-derived featuresfeature18 and feature45 (each + 0.04). Eleven complementary features collectively contributed + 0.1 mean SHAP value.

Notably, the SHAP dependency swarm plot (Fig. 8B) uncovered directional heterogeneity:

- High feature6 values (red) clustered in positive SHAP regions (+ 0.05), intensifying risk predictions.
- feature45 (wavelet-HLL_glcm_ClusterShade_RC) showed dual behavior: high values (red) suppressed predictions (SHAP < − 0.05).
- feature19 values correlated linearly with SHAP magnitude (+ 0.02 to + 0.05), confirming its role in encoding spatial homogeneity.

## Discussion

The findings of this study indicate that the machine learning models developed for analyzing TMJ MRI data exhibit a high level of diagnostic accuracy in identifying ADD and further classifying it into its subtypes: ADDwR and ADDwoR. These machine learning models hold great potential to enhance the diagnostic process for TMDs, thereby enabling more precise diagnoses and facilitating the development of more tailored management strategies. Given that repositioning surgery is the primary treatment for ADDwoR, the accurate identification of such patients is of significant clinical importance.Accurate diagnosis of ADD is essential for effective TMD management, but it remains a challenge for dentists, physicians, and radiologists using traditional imaging methods. Although ADD is the most common asymptomatic form of TMD, it significantly increases the risk of TMJ osteoarthritis, leading to pain, impaired masticatory function, and even TMJ locking [19, 20]. Given the complexity of TMJ imaging, the use of ML-based models offers a promising solution to improve diagnostic accuracy. This study is distinct from prior research as it emphasizes the use of radiomics and ML models for ADD diagnosis, particularly by extracting relevant features from MRI images to enhance classification performance. Unlike earlier studies that applied ML broadly to diagnose TMDs, the present study utilized a more refined image segmentation strategy. The diagnosis of disc displacement primarily depends on the morphological characteristics and signal features of the disc, as well as the relative relationship between the disc and the condyle. For the purpose of this study, the region of interest (ROI) is primarily focused on the structure of the TMJ disc and the condyle, as shown in the sagittal plane.Radiomic characteristics encapsulate both spatial and frequency information. The observed traits may correlate with alterations in tissue density and morphology.The radiomic features extracted in this study. In the case of anterior displacement of the disc, the extracted spatial features may reflect changes in the relative positional structure of the articular disc and the condyle. Particularly, the texture-based features from the GLCM and the GLSZM may reflect underlying structural alterations at the collagen fiber level. Disruption of collagen fiber bundles can manifest as changes in the disc’s internal texture, which may be detected in MRI scans through variations in signal intensity and homogeneity. Specifically, features such as entropy, kurtosis, and grey-level non-uniformity may correspond to the heterogeneity introduced by collagen fiber disruption, with more pronounced changes observed in cases of displacement. This approach excluded irrelevant surrounding tissues, likely contributing to improved diagnostic accuracy.

The integrated LASSO-CV and SHAP framework establishes a robust radiomic signature for preoperative risk stratification in TMJ disc displacement. Multi-scale wavelet features constituted the majority of predictive variables (60% of selected features), with feature18 (wavelet-HLL_gldm_GrayLevelNonUniformity_RO) and feature19 (wavelet-HLH_glcm_Imc1_RO) demonstrating prominent importance based on both LASSO coefficients (Fig. 7B: +0.514/+0.526) and SHAP values (Fig. 8A: +0.04 each). These features likely reflect underlying disorganization of the fibrocartilaginous extracellular matrix, as indicated by increased gray-level non-uniformity and altered spatial correlation patterns—hallmarks of degenerative changes in the TMJ disc. The stability of these features across regularization paths (Fig. 7D, λ > 0.0008) further supports their potential relevance to fibrocartilage structural integrity.Feature49, which received a strong negative coefficient from LASSO (–0.996), was interpreted by SHAP analysis as being associated with reduced risk predictions when its values were low (blue, Fig. 8B). This may correspond to areas of myxoid degeneration, inflammatory infiltration, or early fibrotic remodeling—common pathological changes in displaced TMJ discs. Moreover, SHAP analysis revealed nonlinear relationships not captured by LASSO’s linear weighting; for instance, feature45 exhibited a bidirectional effect, where high values were linked to reduced risk while low values contributed to elevated risk, highlighting the context-dependent nature of radiomic biomarkers.The model’s resilience (MSE: 0.1566 ± 0.0098) can be attributed in part to the predominance of weakly correlated features (Fig. 7A, > 70% with |r| < 0.3). In cases of high collinearity (e.g., feature3 and feature6, *r* = 0.96), LASSO’s L1 regularization effectively eliminated redundancy. SHAP subsequently confirmed the greater discriminative relevance of feature6 via its higher mean absolute SHAP value (+ 0.05), whereas feature3 was excluded from the final importance ranking (Fig. 8A).

In recent years, the integration of ML and AI into medical diagnostics has made substantial progress. Recent studies have highlighted the advantages of ML over traditional clinical methods in diagnosing TMDs using MRI [21, 22]. For instance, some studies have explored computer-aided provisional diagnostic systems using ML for various medical conditions, including MRI-based diagnosis of TMDs [23, 24]. Modern ML algorithms have demonstrated the ability to analyze complex patterns in clinical data, imaging studies, and patient histories with remarkable precision and efficiency [25, 26]. This progress has been facilitated by the growing availability of large datasets, as well as advancements in computational capabilities and algorithms, enabling the development of more sophisticated models. A recent meta-analysis of 28 studies summarized the applications of AI in the diagnosis of TMDs, including both ML and deep learning techniques [27]. However, only a limited number of models have achieved satisfactory diagnostic accuracy. For example, in classifying degenerative joint disease, classifiers such as SVM, RF, and LR have shown favorable results, with some achieving a sensitivity as high as 100% [28, 29]. In the study by Orhan et al. [28], disc displacement was classified into normal and abnormal categories using MRI images, and model performance was assessed based on sensitivity, specificity, and ROC curves. Among the ML models tested, the K-Nearest Neighbors (KNN) classifier was the most effective, achieving an AUC of 0.74 in the validation cohort. The models (KNN, DT, SVM, LR, and RF) tested in the present study showed higher AUCs (0.801–0.874) than those observed by Orhan in the validation cohort. It may be related to the selected ROI. In the study by Orhan, the researchers directly outlined the edges of the joint disc, which is merely a 2D structure.In this study, 3D-Slicer was employed to manually delineate the intervertebral discs and condyles. Clinically, the diagnosis of TMDs is frequently based on observing changes in the geometric relationships of the TMJ between closed and open mouth states. Although there is currently no research substantiating that ML can diagnose TMDs through the geometric relationships of relevant anatomical structures, it is hypothesized that the diagnosis of disc displacement may be facilitated by analyzing the structural relationships among these components. Consequently, when the analysis is restricted to pertinent structural elements, it is anticipated that the classification outcomes derived from ML algorithms will exhibit greater accuracy. These variables could potentially impact the results of image classification. Magnetic resonance segmentation of the TMJ disc is inherently limited by both resolution constraints and the anatomical proximity of adjacent tissues. The inclusion of these surrounding tissues in the segmentation process may affect the radiomic features extracted, potentially capturing variations that are not directly related to the TMJ disc but rather to neighboring structures. This methodology enhances segmentation accuracy, thereby addressing potential confounding factors. Furthermore, the utilization of 3D MRI data, rather than relying exclusively on 2D slices, could enhance the assessment of directional dependencies. Volumetric analysis would furnish a more comprehensive representation of the TMJ disc’s structure, thereby mitigating the risk of variability associated with orientation.Studies have shown that the results of image segmentation and the delineation of ROIs can affect the performance of models in TMDs diagnosis [30, 31].Therefore, the presentstudyused a 3D ROI to more accuratelyinclude the desired Structural information and remove the surroundingirrelevant structural images. The precision of thisstepisdeemed a criticaldeterminant for the subsequent classification of TMDs. In addition, the performance of ML modelsishighlydependentupon the datasets. Future studiesshould compare differentmodelsusing multiple datasets.

In this study, we developed and validated a fusion-phase radiomics model for predicting the anterior displacement of the disc in temporomandibular joint disorders, demonstrating superior performance compared to traditional open and closed phase models. Through a comprehensive analysis of radiomic features extracted from both open and closed phases, our integrated model successfully captured the complementary imaging characteristics inherent to both phases. Five ML models (normal vs. abnormal groups) exhibited enhanced performance within the fusion model relative to the individual open and closed models, with the fused model achieving scores ranging from 0.801 to 0.874, the open model from 0.801 to 0.829, and the closed model from 0.774 to 0.828. In the subgroup with additional diagnoses of ADDwR and ADDwoR, the fused model again outperformed the open and closed models in the test cohort, with scores of 0.699 to 0.712 for the fused model, 0.655 to 0.689 for the open model, and 0.669 to 0.697 for the closed model. The stepwise improvement in predictive performance from the open and closed models to the fused model underscores the value of integrating complementary information from images captured during both mouth positions. This finding aligns with the complex pathophysiology of TMDs, wherein the diagnosis of such disorders necessitates the synthesis of information derived from imaging conducted in both open and closed positions. A noteworthy finding was the superior and most stable performance of LR on the fused features. This phenomenon is likely attributed to three interrelated factors: (1) The fused feature space, while discriminative, was of moderate dimensionality and rigorously filtered through feature selection; (2) LR combined with L1/L2 regularization effectively suppresses overfitting—a critical advantage given our cohort size; (3) In contrast, tree-based ensembles (RF, XGBoost, LightGBM), despite their inherent flexibility, exhibited greater sensitivity to training data perturbations and potential marginal overfitting without aggressive regularization. This sensitivity was observed in their slightly higher performance variance across validation folds compared to the test set, particularly in feature spaces with correlated predictors. The parametric simplicity and linearity of LR further enhance model interpretability, aligning with clinical deployment needs.

Unlikepreviousstudies [25, 26], this research further differentiated between ADDwR and ADDwoR using machine learning models. Discriminating between the two ADD types is clinically meaningful because of the differences in management between the two entities, ADDwR being mostly managed conservatively and ADDwoR being an indication of repositioning surgery [32]. Furthermore, patients with ADDwR and those with ADDwoR exhibit significant differences in the risk of developing TMJ osteoarthritis. Compared to patients with ADDwR, those with ADDwoR are approximately three times more likely to develop TMJ osteoarthritis [33]. In evaluating the ADDwR and ADDwoR classification models, the AUCs were lower than those for the normal/abnormal classification. This discrepancy may be attributed to the relatively small sample sizes for the ADDwR and ADDwoR groups. This innovation holds great potential for improving treatment strategies and predicting patient prognosis [32]. The high diagnostic accuracy achieved by the model represents a significant advancement in the non-invasive diagnosis of TMDs and could reduce the need for invasive diagnostic procedures [34].

AI can support clinicians in providing more accurate and timely diagnoses, ultimately improving patient outcomes. Additionally, the integration of AI into diagnostic workflows could alleviate the workload of healthcare professionals, allowing them to focus on complex cases that require human expertise. However, further research is needed to assess the generalizability of these findings across diverse populations and healthcare settings. The development of more sophisticated ML models capable of integrating multimodal data (clinical, imaging, and genetic) could further enhance diagnostic performance in TMDs. Moreover, addressing ethical considerations and establishing robust regulatory frameworks are essential to ensure patient safety and data privacy in the use of AI in healthcare [35, 36]. As AI technology evolves, fostering interdisciplinary collaboration among clinicians and data scientists will be critical to fully harnessing the potential of ML in diagnosing and managing TMDs.

Our radiomics framework and deep learning approaches represent synergistic yet distinct paradigms for TMJ diagnosis. While DL models automate the detection of disc morphology and spatial relationships, they often function as “black boxes” with limited clinical interpretability. In contrast, our method extracts handcrafted features from open- and closed-mouth phases to quantify dynamic condylar trajectory and disc deformation—parameters directly linked to joint biomechanics and readily actionable for clinical staging (e.g., ADDWR vs. ADDWoR). Future studies should explore hybrid frameworks: for example, integrating DL-based segmentation of disc-condyle complexes with radiomic kinetics features to build interpretable predictive models. Such integration could leverage DL’s robustness to image noise and radiomics’ strength in feature transparency, ultimately enabling precision medicine for TMDs.

The present study had several limitations. First, the radiomics approach employed in this study relies on explicitly defined, handcrafted features, which provide greater interpretability compared with the “black box” nature of DLmodels. However, the present work represents a preliminary investigation primarily aimed at validating this conventional methodology. As a result, the potential synergistic advantages of integrating radiomics with DL techniques were not explored and should be addressed in future studies. Second, the generalizability of our findings is limited by the use of a single-center dataset and the absence of external validation with multi-center. In addition, the relatively small sample sizes of the ADDwR and ADDwoR subgroups reduce the robustness of the subgroup analyses. This limitation is particularly reflected in the perfect sensitivity (1.000) achieved by certain classifiers in the validation cohort; although promising, these results should be interpreted with caution due to the restricted validation set size and require confirmation in larger, multi-center external validation studies.Future prospective studies should aim to improve study design in patient selection, index tests, and reference standards, with the goal of identifying more accurate and precise ML methods.While the single-center design and scanner homogeneity enhance internal validity, they inevitably constrain model generalizability. To bridge this translational gap, our future work will include establishing a multicenter consortium (≥ 3 institutions) using diverse scanners (Siemens/GE/Philips) and acquisition parameters (e.g., 1–5 mm slice thickness), with stratified sampling of ≥ 500 patients to capture population heterogeneity.While our pipeline automates feature analysis and classification, the current workflow requires manual ROI segmentation by an expert radiologist. This step presents a bottleneck for fully automated clinical deployment and may impact scalability. Future work will integrate deep learning-based segmentation (e.g., nnU-Net or 3D U-Net architectures) to eliminate this manual dependency. For seamless clinical deployment, the model could be implemented as a lightweight plugin within existing Picture Archiving and Communication Systems (PACS) or Radiology Information Systems (RIS) in the future.

## Conclusions

In conclusion, this study developed machine learning models using five classifiers to analyze TMJ MRI data within a fusion model, effectively classifying TMJ ADD into normal and abnormal categories. Additionally, the study introduced a novel application of machine learning to further distinguish abnormal cases into ADDwR and ADDwoR. Although the results are promising, further research is needed to refine these models and improve their clinical utility for the diagnosis and management of TMDs.

## Supplementary Information

Supplementary Material 1.

## Data Availability

All data generated or analysed during this study are included in this published article.

## Abbreviations

ADD: Anterior disc displacement
ADDwR: Anterior disk displacement with reduction
ADDwoR: Anterior disk displacement without reduction
DT: Decision tree
LR: Logistic regression
ML: Machine learning
MRI: Magnetic resonance imaging
KNN: K-nearest neighbors
RF: Random forest
SVM: Support vector machines
TMDs: Temporomandibular disorders
TMJDs: Temporomandibular joint disorders
TMJ: Temporomandibular joint
TMJDD: Temporomandibular joint disc displacement
