# Supplementary material for: Semi-automatic detection of anteriorly displaced temporomandibular joint discs in magnetic resonance images using machine learning
Source: BMC Oral Health. 2025 Oct 10;25:1591. doi: 10.1186/s12903-025-06981-5 (PMC12513056; doi:10.1186/s12903-025-06981-5)
Supplement: Supplementary file 1 — Supplementary Material 1. [file 12903_2025_6981_MOESM1_ESM.docx]

**Supplementary S1.**

**Part 0. MRI Acquisition Parameters**

For T2WI, the imaging parameters were as follows: repetition time/echo time (TR/TE) of 2740.0/68.0 ms, number of excitations (NEX) of 4, field of view (FOV) of 16×16 cm, bandwidth of 35.71, slice thickness/spacing of 2.0/0.5 mm, matrix size of 288×256, and scan time of 2 minutes 39 seconds. For proton density-weighted imaging (PDWI), the parameters were: TR/TE of 1951.0/35.0 ms, NEX of 4, FOV of 16×16 cm, bandwidth of 35.71, slice thickness/spacing of 2.0/0.5 mm, matrix size of 288×224, and scan time of 2 minutes 24 seconds. This standardized protocol was used throughout the study period to ensure consistent and precise detection of TMJ disc displacements.

**Part 1. Radiomic Feature Extraction**

We analyzed the radiomic features by adopting the V3.0.1 version of Pyradiomics. The images were normalized with a normalization ratio of 50, and the BSpline interpolator method was used to resample all images to the average spacing of training set. Radiomic features we extracted included first order statistics, shape-based (2D and 3D), Gray Level cooccurence matrix (GLCM), Gray Level Size Zone Matrix (GLSZM), Gray Level Run Length Matrix (GLRLM), Neighbouring Gray Tone Difference Matrix (NGTDM), Gray Level Dependence Matrix (GLDM). All the above features were extracted using the default settings. In addition, there are Laplacian of Gaussianfiltered (with Sigma values 1.0, 3.0, 5.0), wavelet-decomposition-based (using the coiflet 1 function). A specific description of the feature can be found in the Pyradiomics document. For each patient, we extracted 1130 features.

This is the PyRadiomics configuration for the radiomics experiment setup in this study:

# Settings to use, possible settings are listed in the documentation (section "Customizing the extraction").

setting:

normalize: True

normalizeScale: 50

binWidth: 25

label: 1

interpolator: 'sitkBSpline'

resampledPixelSpacing: [x_train_average spacing,y_train_average spacing,z_train_average spacing]

imageType:

Original: {}

Wavelet: {}

LoG:

sigma: [1., 3., 5.]

featureClass:

shape: #all shape

firstorder: # specifying an empty list has the same effect as specifying nothing.

glcm: # Disable SumAverage by specifying all other GLCM features available

glrlm: # for lists none values are allowed, in this case, all features are enabled

glszm:

gldm: # contains deprecated features, but as no individual features are specified, the deprecated features are not enabled

ngtdm:

voxelSetting:

kernelRadius: 2

maskedKernel: true

initValue: 0

voxelBatch: 10000

**Part 2. Feature Selection**

We initiated the process by calculating the F-values between the labels and features using the f_classif function from the feature selection library. Subsequently, we employed the SelectKBest function from the same library with k set to 100 to select the top 100 features. Following this, we calculated the chi-squared values for the features using the chi2 function from the feature selection library and applied SelectKBest again, this time with k=50, to further narrow down the features to the top 50. Finally, we refined these 50 features using the Least Absolute Shrinkage and Selection Operator (LASSO) with cross-validation. LASSO, implemented via LassoCV from scikit-learn, integrates L1 regularization into its loss function to manage model complexity and automatically selects features by shrinking the coefficients of irrelevant features to zero. The optimal regularization parameter (alpha) was determined through 10-fold cross-validation and only features with non-zero coefficients were retained in the final feature set.The regularization path was systematically constructed using np.logspace(-4, 4, 300) to generate 300 α values spanning four orders of magnitude, ensuring comprehensive coverage of potential regularization intensities from minimal shrinkage to complete feature elimination. This configuration adheres to the CRISP-ML standard for hyperparameter optimization in high-dimensional radiomic studies.

**Part 3. Model Construction**

The study group was randomly divided into training and validation cohorts at a ratio of 8:2. The training cohort was standardized using the preprocessing.StandardScaler() function, and the validation cohort was standardized using the parameters derived from the training cohort. The hyperparameters for each classifier used in the model training were set as follows:

def class_para(self):

# SVM

self.svm_kernel = ['rbf', 'sigmoid']

# self.svm_kernel = ['linear', 'poly', 'rbf', 'sigmoid']

self.svm_C = [1e-8, 1e-6, 1e-5, 1e-3, 1e-2, 1e-1, 1, 32, 100, 200, 300, 400, 1000]

self.svm_Gamma = [1e-8, 1e-6, 1e-5, 1e-3, 1e-2, 1e-1]

self.svm_classwight= [{0: 0.55, 1: 1}, {0: 0.6, 1: 1}, {0: 0.65, 1: 1}, {0: 0.7, 1: 1}, {0: 0.8, 1: 1},{0: 1, 1: 1}]

self.svm_max_iter = [2, 4, 10, 20, 25, 30, 40, 60, 140, 200, 300, -1]

self.svm_grid_search_param= [{"kernel": self.svm_kernel, "C": self.svm_C, "gamma": self.svm_Gamma, "class_weight": self.svm_classwight, "max_iter": self.svm_max_iter}]

# Logistic

self.lr_penalty = ['l2']

self.lr_C = [0.01, 0.05, 0.1, 0.3, 0.4, 0.5, 0.6, 0.8, 1.0, 1.2]

self.lr_max_iter = [10, 20, 40, 60, 80, 100, 150, 200, 400, 500]

self.lr_tol = [1e-5, 1e-4, 1e-3, 1e-2]

self.lr_grid_search_param= [{"penalty": self.lr_penalty, "C": self.lr_C, "max_iter": self.lr_max_iter, "tol": self.lr_tol}]

#RF

self.rf_n_estimators = [10, 20,30 , 50 ,60 ,80 ,101, 151, 201, 251, 301, 351, 401, 451, 501, 551, 800]

self.rf_max_depth=[ None ,1, 3, 4, 5, 6, 8, 10, 12, 15, 20]

self.rf_min_samples_split=[1, 3, 5, 7,9 , 10, 12, 15]

self.rf_min_samples_leaf: [1, 2, 3, 4, 5, 7, 9, 10],

self.rf_grid_search_param= [{"n_estimators": self.rf_n_estimators, " max_depth ": self.rf_max_depth , "min_samples_split ": self.rf_min_samples_split , "min_samples_leaf": self.rf_min_samples_leaf}]

# DT

self.dt_criterion = ['gini', 'entropy']

self.dt_max_depth = [None, 5, 8, 10, 12, 15, 17, 20, 25, 30]

self.dt_min_samples_split = [2, 3, 5, 7, 10]

self.dt_min_samples_leaf = [1, 2, 3, 4, 5, 7, 9, 10]

self.dt_grid_search_param= [{"criterion": self.dt_criterion, "max_depth": self.dt_max_depth, "min_samples_split": self.dt_min_samples_split, "min_samples_leaf": self.dt_min_samples_leaf}]

# KNN

self.knn_n_neighbors = [3, 5, 7, 10, 15, 20]

self.knn_weights = ['uniform', 'distance']

self.knn_metric = ['euclidean', 'manhattan']

self.knn_grid_search_param= [{"n_neighbors": self.knn_n_neighbors, "weights": self.knn_weights, "metric": self.knn_metric}]

**Supplementary Table S1.**Assessment of diagnostic performance using five indicators for normal and abnormal classifications in the Fusion Model across both training and validation cohorts.

| Variable | AUC (95% CI) | F1 score | Sensitivity | Specificity | Precision | P ^a^ |
| --- | --- | --- | --- | --- | --- | --- |
| Training cohort (n=305) |  | | | | |  |
| DT | 0.854 (0.814-0.894) | 0.784（0.738-0.830） | 0.881（0.845-0.917） | 0.713（0.662-0.763） | 0.707（0.656-0.758） | <0.0001 |
| KNN | 0.847 (0.807-0.887) | 0.754  （0.706-0.802） | 0.791  (0.745-0.837) | 0.76  (0.712-0.808) | 0.721  (0.671-0.771) | <0.0001 |
| LR | 0.899 (0.865-0.933) | 0.806  (0.762-0.85) | 0.836  (0.794-0.878) | 0.813  (0.769-0.857) | 0.778  (0.731-0.825) | <0.0001 |
| RF | 0.889 (0.854-0.924) | 0.808  (0.7640.852) | 0.881  (0.847-0.917) | 0.766  (0.718-0.814) | 0.747  (0.698-0.796) | <0.0001 |
| SVM | 0.890 (0.855-0.925) | 0.788  (0.742-0.834) | 0.776  (0.729-0.823) | 0.848  (0.808-0.888) | 0.8  (0.755-0.845) | <0.0001 |
| Validation cohort (n=77) |  | | | | |  |
| DT | 0.801 (0.712-0.890) | 0.775  (0.682-0.868) | 0.912  (0.848-0.975) | 0.651  (0.545-0.757) | 0.674  (0.569-0.779) | <0.0001 |
| KNN | 0.852 (0.773-0.931) | 0.829  (0.745-0.913) | 1.000  (1.0-1.0) | 0.674  (0.569-0.779) | 0.708  (0.606-0.81) | <0.0001 |
| LR | 0.841 (0.759-0.923) | 0.84  (0.758-0.922) | 1.000  (1.0-1.0) | 0.698  (0.595-0.801) | 0.723  (0.623-0.823) | <0.0001 |
| RF | 0.874 (0.799-0.948) | 0.819  (0.733-0.905) | 1.000  (1.0-1.0) | 0.651  (0.545-0.757) | 0.694  (0.591-0.797) | <0.0001 |
| SVM | 0.863 (0.786-0.939) | 0.81  (0.722-0.898) | 1.000  (1.0-1.0) | 0.628  (0.520-0.736) | 0.680  (0.576-0.784) | <0.0001 |

SVM: support vector machine; LR: logistic regression; RF: random forest; decision tree: DT; K-nearest neighbors: KNN ; CI, confidence interval; AUC, area under the receiver operating characteristic curve. ^a^ P value is the significance level for comparing the AUC with a random case (AUC=0.5)

**Supplementary Table S2.**Assessment of diagnostic performance using five indicators for normal and abnormal classifications in the open phase across both training and validation cohorts.

| Variable | AUC (95% CI) | F1 score | Sensitivity | Specificity | Precision | P ^a^ |
| --- | --- | --- | --- | --- | --- | --- |
| Training cohort (n=305) |  | | | | |  |
| DT | 0.772 (0.725- 0.819) | 0.743  (0.694-0.792) | 0.925  (0.895-0.955) | 0.556  (0.5-0.612) | 0.620  (0.566-0.674) | <0.0001 |
| KNN | 0.834(0.792-0.876) | 0.736  (0.687-0.786) | 0.769  (0.722-0.816) | 0.749  (0.7-798) | 0.705  (0.654-0.756) | <0.0001 |
| LR | 0.854 (0.814- 0.894) | 0.786  (0.74-0.832) | 0.903  (0.87-0.936) | 0.690  (0.638-0.742) | 0.695  (0.643-0.747) | <0.0001 |
| RF | 0.842(0.801-0.883) | 0.750  (0.701-0.799) | 0.806  (0.762-0.85) | 0.731  (0.681-0.781) | 0.701  (0.65-0.752) | <0.0001 |
| SVM | 0.839 (0.798-0.880) | 0.766  (0.718-0.814) | 0.881  (0.845-0.917) | 0.673  (0.62-0.726) | 0.678  (0.626-0.73) | <0.0001 |
| Validation cohort (n=77) |  | | | | |  |
| DT | 0.774(0.712-0.890) | 0.782  (0.69-0.874) | 1  (1.0-1.0) | 0.558  (0.447-0.669) | 0.642  (0.535-0.749) | <0.0001 |
| KNN | 0.818(0.735-0.907) | 0.791  (0.7-0.882) | 1  (1.0-1.0) | 0.581  (0.471-0.691) | 0.654  (0.548-0.76) | <0.0001 |
| LR | 0.828 (0.731-0.903) | 0.805  (0.717-0.893) | 0.971  (0.934-1.0) | 0.651  (0.545-0.757) | 0.688  (0.585-0.791) | <0.0001 |
| RF | 0.808 (0.745-0.913) | 0.790  (0.699-0.881) | 0.941  (0.888-0.994) | 0.651  (0.545-0.757) | 0.681  (0.577-0.785) | <0.0001 |
| SVM | 0.808(0.733- 0.905) | 0.716  (0.615-0.817) | 0.706  (0.604-0.808) | 0.791  (0.7-0.882) | 0.727  (0.627-0.827) | <0.0001 |

SVM: support vector machine; LR: logistic regression; RF: random forest; decision tree: DT ; K-nearest neighbors: KNN ; CI, confidence interval; AUC, area under the receiver operating characteristic curve. ^a^ P value is the significance level for comparing the AUC with a random case (AUC=0.5)

**Supplementary Table S3.**Assessment of diagnostic performance using five indicators for normal and abnormal classifications in the closed phase across both training and validation cohorts.

| Variable | AUC (95% CI) | F1 score | Sensitivity | Specificity | Precision | P ^a^ |
| --- | --- | --- | --- | --- | --- | --- |
| Training cohort (n=305) |  | | | | |  |
| DT | 0.833 (0.791-0.875) | 0.760  (0.712-0.808) | 0.873  (0.836-0.91) | 0.667  (0.614-0.72) | 0.672  (0.619-0.725) | <0.0001 |
| KNN | 0.853 (0.813-0.893) | 0.782  (0.736-0.828) | 0.843  (0.802-0.884) | 0.754  (0.706-0.802) | 0.729  (0.679-0.779) | <0.0001 |
| LR | 0.853 (0.813-0.893) | 0.74  (0.691-0.789) | 0.731  (0.681-0.781) | 0.807  (0.763-0.851) | 0.748  (0.669-0.797) | <0.0001 |
| RF | 0.852 (0.812-0.892) | 0.741  (0.692-0.79) | 0.791  (0.745-0.837) | 0.731  (0.681-0.781) | 0.697  (0.645-0.749) | <0.0001 |
| SVM | 0.862(0.823-0.901) | 0.789  (0.743-0.835) | 0.896  (0.862-0.93) | 0.708  (0.657-0.759) | 0.706  (0.655-0.757) | <0.0001 |
| Validation cohort (n=77) |  | | | | |  |
| DT | 0.801(0.712-0.890) | 0.759  (0.663-0.855) | 0.971  (0.934-1.008) | 0.535  (0.424-0.646) | 0.623  (0.515-0.731) | <0.0001 |
| KNN | 0.821 (0.735-0.907) | 0.782  (0.69-0.874) | 1  (1.0-1.0) | 0.558  (0.447-0.669) | 0.642  (0.535-0.749) | <0.0001 |
| LR | 0.817 (0.730-0.903) | 0.821  (0.735-0.907) | 0.941  (0.888-0.994) | 0.721  (0.621-0.821) | 0.727  (0.627-0.827) | <0.0001 |
| RF | 0.829 (0.745-0.913) | 0.8  (0.711-0.889) | 1  (1.0-1.0) | 0.605  (0.496-0.714) | 0.667  (0.562-0.772) | <0.0001 |
| SVM | 0.819 (0.733-0.905) | 0.712  (0.611-0.813) | 0.618  (0.509-0.727) | 0.907  (0.842-0.972) | 0.84  (0.758-0.922) | <0.0001 |

SVM: support vector machine; LR: logistic regression; RF: random forest; decision tree: DT ; K-nearest neighbors: KNN ; CI, confidence interval; AUC, area under the receiver operating characteristic curve. ^a^ P value is the significance level for comparing the AUC with a random case (AUC=0.5)

**Supplementary Table S4.**Assessment of diagnostic performance using five indicators for the ADDwR and ADDwoR classifications in the Fusion Model across both training and validation cohorts.

| Variable | AUC (95% CI) | F1 score | Sensitivity | Specificity | Precision | P ^a^ |
| --- | --- | --- | --- | --- | --- | --- |
| Training cohort (n=134) |  | | | | |  |
| DT | 0.732 (0.657-0.807) | 0.806  (0.739-0.873) | 0.843  (0.781-0.905) | 0.511  (0.426-0.596) | 0.773  (0.702-0.844) | <0.0001 |
| KNN | 0.733 (0.658-0.808) | 0.804  (0.737-0.871) | 0.809  (0.742-0.876) | 0.6  (0.517-0.683) | 0.8  (0.732-0.868) | <0.0001 |
| LR | 0.747 (0.673-0.821) | 0.707  (0.63-0.784) | 0.596  (0.513-0.679) | 0.822  (0.7570.888) | 0.869  (0.812-0.926) | <0.0001 |
| RF | 0.733 (0.658-0.808) | 0.8  (0.732-0.868) | 0.809  (0.742-0.876) | 0.578  (0.494-0.662) | 0.791  (0.722-0.86) | <0.0001 |
| SVM | 0.731 (0.656-0.806) | 0.776  (0.705-0.847) | 0.719  (0.643-0.795) | 0.733  (0.658-0.808) | 0.842  (0.78-0.904) | <0.0001 |
| Validation cohort (n=34) |  | | | | |  |
| DT | 0.699 (0.545-0.853) | 0.783  (0.644-0.922) | 0.818  (0.688-0.948) | 0.5  (0.332-0.668) | 0.75  (0.604-0.896) | <0.0001 |
| KNN | 0.712 (0.559-0.864) | 0.686  (0.53-0.842) | 0.545  (0.378-0.712) | 0.917  (0.824-1.01) | 0.923  (0.833-1.013) | <0.0001 |
| LR | 0.705 (0.551-0.858) | 0.791  (0.654-0.928) | 0.773  (0.632-0.914) | 0.667  (0.509-0.825) | 0.81  (0.678-0.942) | <0.0001 |
| RF | 0.693 (0.537-0.848) | 0.581  (0.425-0.747) | 0.409  (0.244-0.574) | 1  (1.0-1.0) | 1  (1.0-1.0) | <0.0001 |
| SVM | 0.701 (0.547-0.855) | 0.606  (0.442-0.77) | 0.455  (0.288-0.622) | 0.917  (0.824-1.01) | 0.909  (0.812-1.006) | <0.0001 |

SVM: support vector machine; LR: logistic regression; RF: random forest; decision tree: DT ; K-nearest neighbors: KNN ; CI, confidence interval; AUC, area under the receiver operating characteristic curve. ^a^ P value is the significance level for comparing the AUC with a random case (AUC=0.5)

**Supplementary Table S5.**Assessment of diagnostic performance using five indicators for the ADDwR and ADDwoR classifications in the open phase across both training and validation cohorts.

| Variable | AUC (95% CI) | F1 score | Sensitivity | Specificity | Precision | P ^a^ |
| --- | --- | --- | --- | --- | --- | --- |
| Training cohort (n=134) |  | | | | |  |
| DT | 0.728 (0.653-0.803) | 0.771  (0.7-0.842) | 0.719  (0.643-0.795) | 0.711  (0.634-0.788) | 0.831  (0.768-0.894) | <0.0001 |
| KNN | 0.719 (0.642-0.795) | 0.825  (0.76-0.889) | 0.899  (0.848-0.95) | 0.444  (0.36-0.528) | 0.762  (0.69-0.834) | <0.0001 |
| LR | 0.712 (0.635-0.788) | 0.701  (0.623-0.779) | 0.607  (0.524-0.69) | 0.756  (0.683-0.829) | 0.831  (0.768-0.894) | <0.0001 |
| RF | 0.736 (0.661-0.810) | 0.807  (0.74-0.874) | 0.82  (0.755-0.885) | 0.578  (0.494-0.662) | 0.793  (0.724-0.862) | <0.0001 |
| SVM | 0.703 (0.625-0.780) | 0.753  (0.68-0.826) | 0.719  (0.6423-0.795) | 0.622  (0.54-0.704) | 0.79  (0.721-0.859) | <0.0001 |
| Validation cohort (n=34) |  | | | | |  |
| DT | 0.676 (0.519-0.833) | 0.816  (0.686-0.946) | 0.909  (0.812-1.006) | 0.417  (0.251-0.583) | 0.741  (0.594-0.888) | <0.0001 |
| KNN | 0.686 (0.529-0.842) | 0.718  (0.567-0.869) | 0.636  (0.474-0.798) | 0.75  (0.604-0.896) | 0.824  (0.696-0.952) | <0.0001 |
| LR | 0.689 (0.533-0.844) | 0.762  (0.619-0.905) | 0.727  (0.577-0.878) | 0.667  (0.509-0.825) | 0.8  (0.666-0.934) | <0.0001 |
| RF | 0.697 (0.542-0.851) | 0.857  (0.739-0.975) | 0.955  (0.885-1.025) | 0.5  (0.332-0.668) | 0.778  (0.638-0.918) | <0.0001 |
| SVM | 0.655 (0.495-0.815) | 0.718  (0.567-0.869) | 0.636  (0.474-0.798) | 0.75  (0.604-0.896) | 0.824  (0.696-0.952) | <0.0001 |

SVM: support vector machine; LR: logistic regression; RF: random forest; decision tree: DT ; K-nearest neighbors: KNN ; CI, confidence interval; AUC, area under the receiver operating characteristic curve. ^a^ P value is the significance level for comparing the AUC with a random case (AUC=0.5)

**Supplementary Table S6.**Assessment of diagnostic performance using five indicators for ADDwR and ADDwoR classifications in the closed phase across both training and validation cohorts.

| Variable | AUC (95% CI) | F1 score | Sensitivity | Specificity | Precision | P ^a^ |
| --- | --- | --- | --- | --- | --- | --- |
| Training cohort (n=134) |  | | | | |  |
| DT | 0.727 (0.652-0.802) | 0.731  (0.656-0.806) | 0.64  (0.559-0.721) | 0.778  (0.708-0.848) | 0.851  (0.791-0.911) | <0.0001 |
| KNN | 0.729 (0.654-0.804) | 0.762  (0.69-0.834) | 0.719  (0.643-0.795) | 0.667  (0.587-0.747) | 0.81  (0.744-0.876) | <0.0001 |
| LR | 0.716 (0.639-0.792) | 0.714  (0.637-0.791) | 0.618  (0.536-0.7) | 0.778  (0.708-0.848) | 0.846  (0.785-0.907) | <0.0001 |
| RF | 0.734 (0.659-0.809) | 0.802  (0.735-0.869) | 0.82  (0.755-0.885) | 0.556  (0.482-0.64) | 0.785  (0.715-0.855) | <0.0001 |
| SVM | 0.699 (0.621-0.777) | 0.662  (0.582-0.742) | 0.551  (0.467-0.635) | 0.778  (0.708-0.848) | 0.831  (0.768-0.894) | <0.0001 |
| Validation cohort (n=34) |  | | | | |  |
| DT | 0.697 (0.542-0.851) | 0.818  (0.688-0.948) | 0.818  (0.688-0.948) | 0.667  (0.509-0.825) | 0.818  (0.688-0.948) | <0.0001 |
| KNN | 0.678 (0.521-0.835) | 0.826  (0.699-0.953) | 0.864  (0.749-0.979) | 0.583  (0.417-0.749) | 0.792  (0.656-0.928) | <0.0001 |
| LR | 0.689 (0.533-0.845) | 0.8  (0.666-0.934) | 0.818  (0.688-0.948) | 0.583  (0.417-0.749)) | 0.783  (0.644-0.922) | <0.0001 |
| RF | 0.689 (0.533-0.845) | 0.8  (0.666-0.934) | 0.818  (0.688-0.948) | 0.583  (0.417-0.749)) | 0.783  (0.644-0.922) | <0.0001 |
| SVM | 0.663 (0.504-0.822) | 0.8  (0.666-0.934) | 0.818  (0.688-0.948) | 0.583  (0.417-0.749)) | 0.783  (0.644-0.922) | <0.0001 |

SVM: support vector machine; LR: logistic regression; RF: random forest;decisiontree:DT;K-nearestneighbors:KNN; CI, confidence interval; AUC, area under the receiver operating characteristic curve. ^a^ P value is the significance level for comparing the AUC with a random case (AUC=0.5)


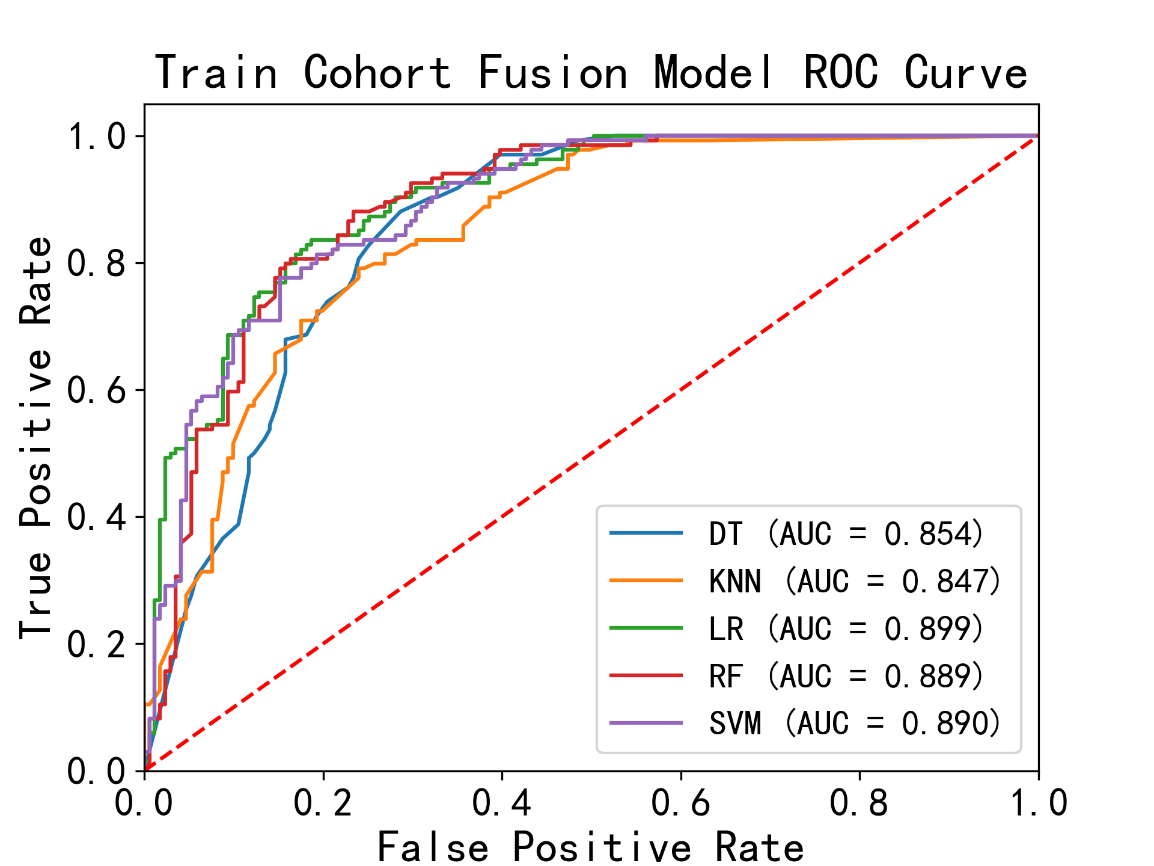


Figure1A


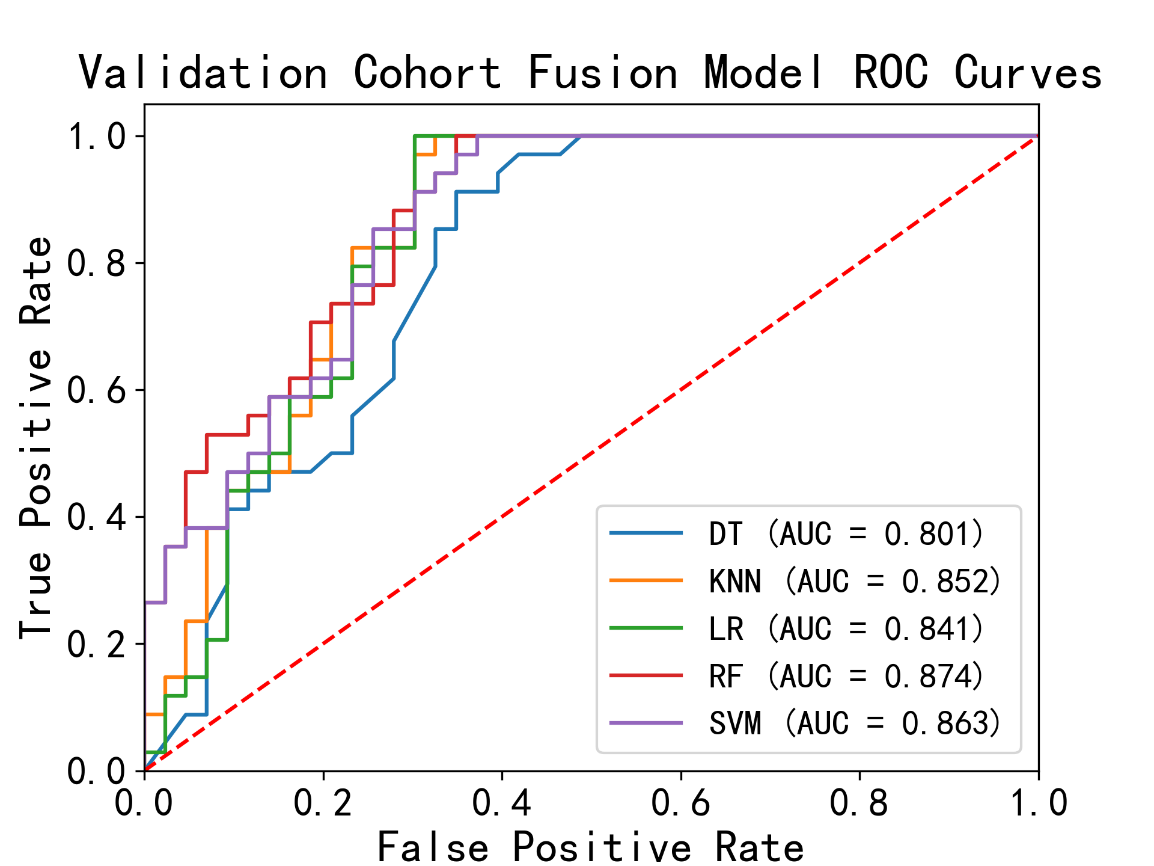


Figure1B

**Supplementary　Figure１.**Diagnostic performance of the five models for classifying normal and abnormal cases using the Fusion Model in the training (A) and validation (B) cohorts. DT: decision tree; KNN: K-nearest neighbors; LR: logistic regression; RF: random forest; SVM: support vector machine; AUC: area under the curve.


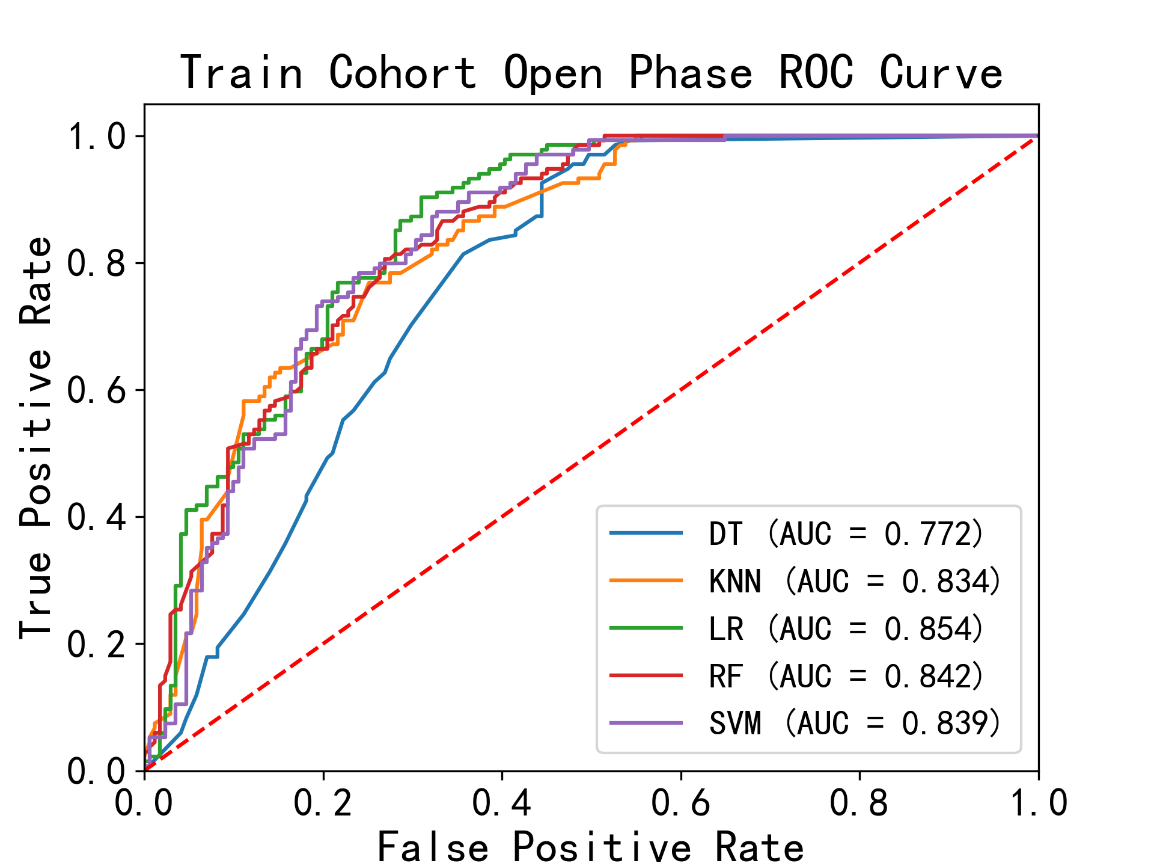


Figure2A


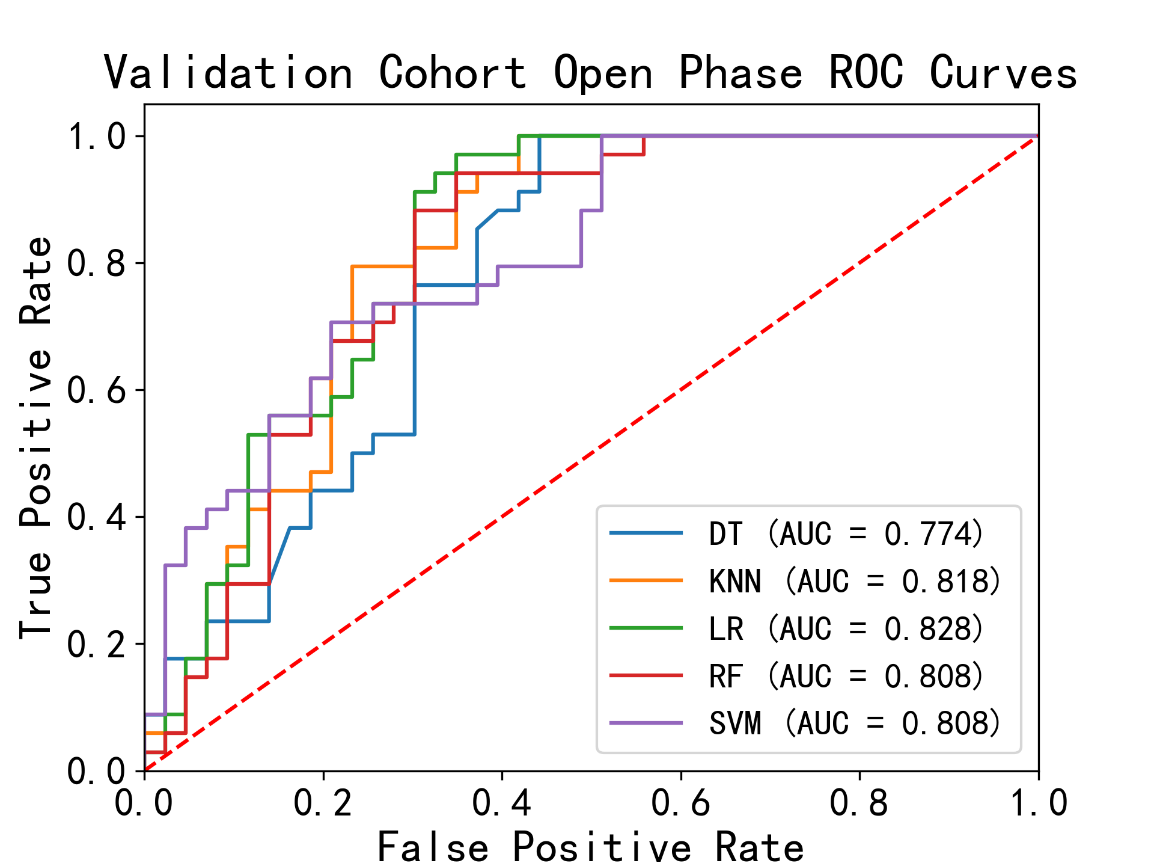


Figure2B

**Supplementary　Figure 2.**Diagnostic performance of the five models for classifying normal and abnormal cases using the open phase in the training (A) and validation (B) cohorts. DT: decision tree; KNN: K-nearest neighbors; LR: logistic regression; RF: random forest; SVM: support vector machine; AUC: area under the curve.


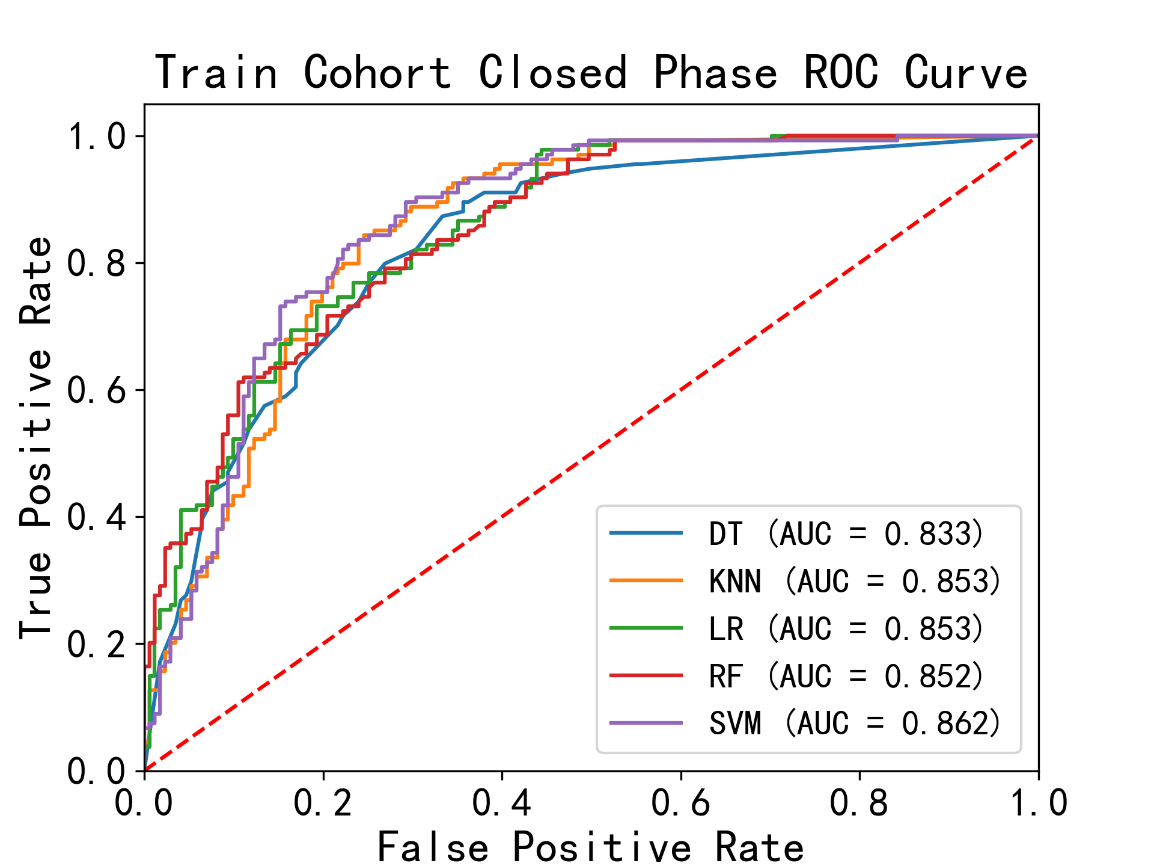


Figure 3A


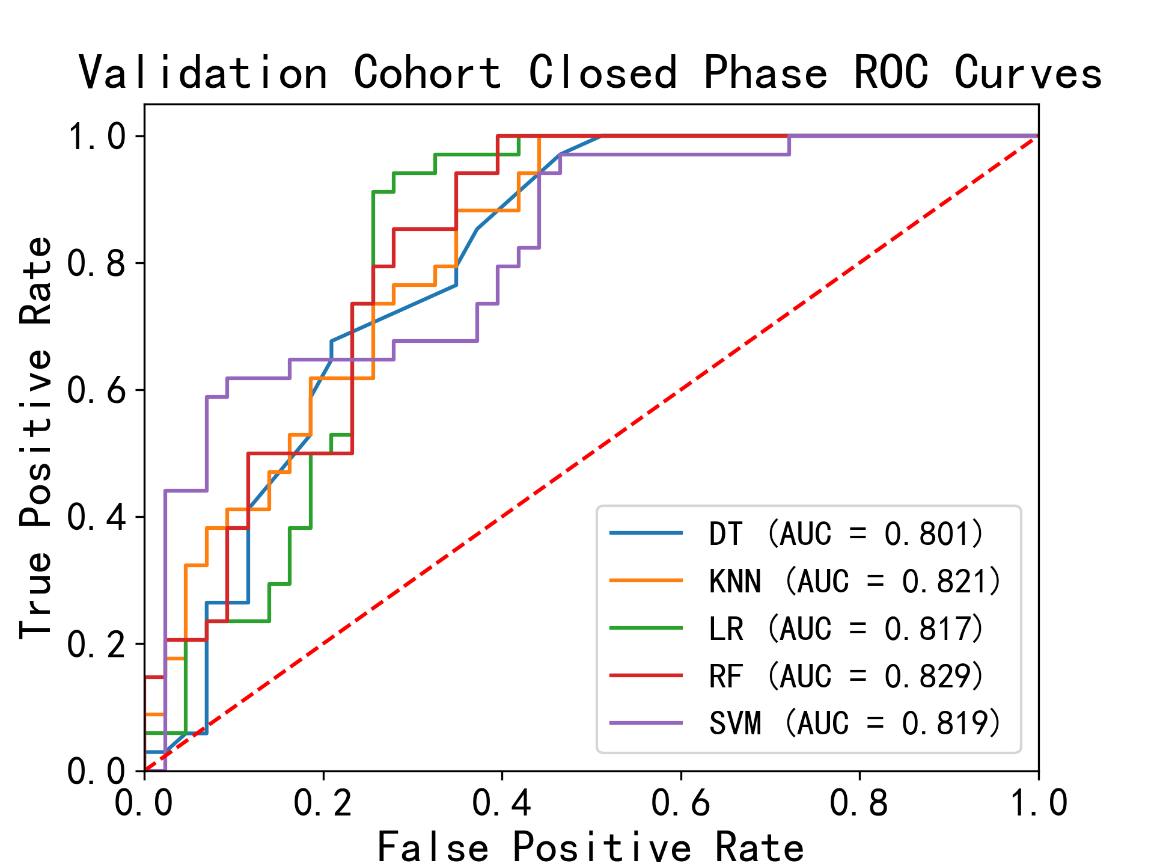


Figure 3B

**Supplementary　Figure 3.**Diagnostic performance of the five models for classifying normal and abnormal cases using the close phase in the training (A) and validation (B) cohorts. DT: decision tree; KNN: K-nearest neighbors; LR: logistic regression; RF: random forest; SVM: support vector machine; AUC: area under the curve.


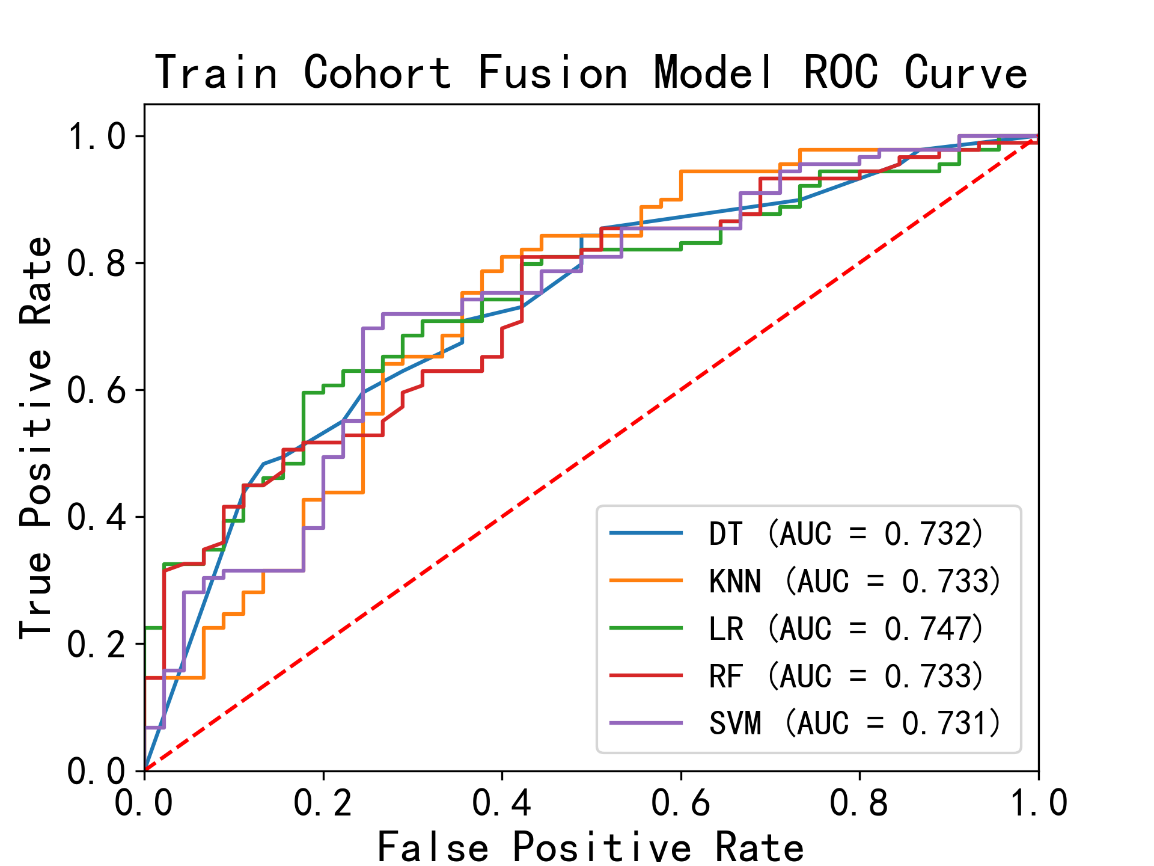


Figure 4A


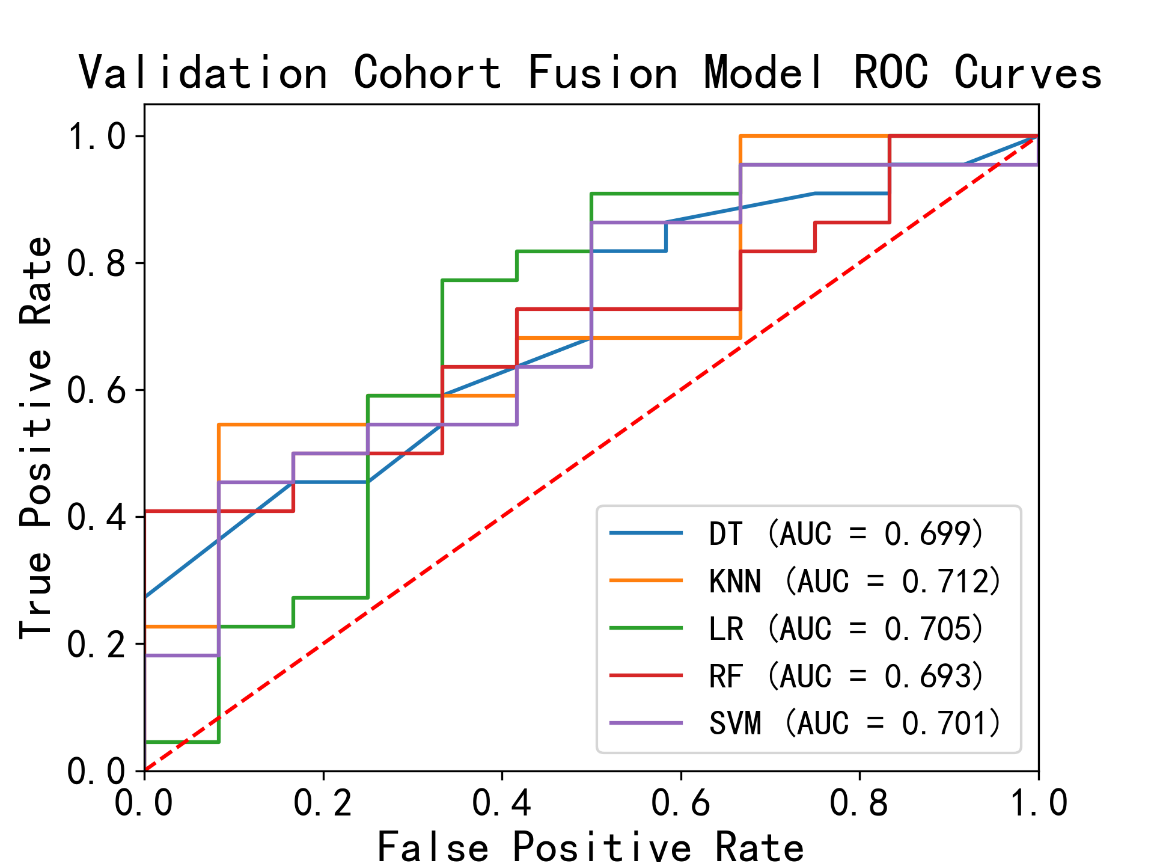


Figure 4B

**Supplementary　Figure 4.**Diagnostic performance of the five models for classifying anterior disc displacement with reduction (ADDwR) and without reduction (ADDwoR) using the Fusion Model in the training (A) and validation (B) cohorts. DT: decision tree; KNN: K-nearest neighbors; LR: logistic regression; RF: random forest; SVM: support vector machine; AUC: area under the curve.


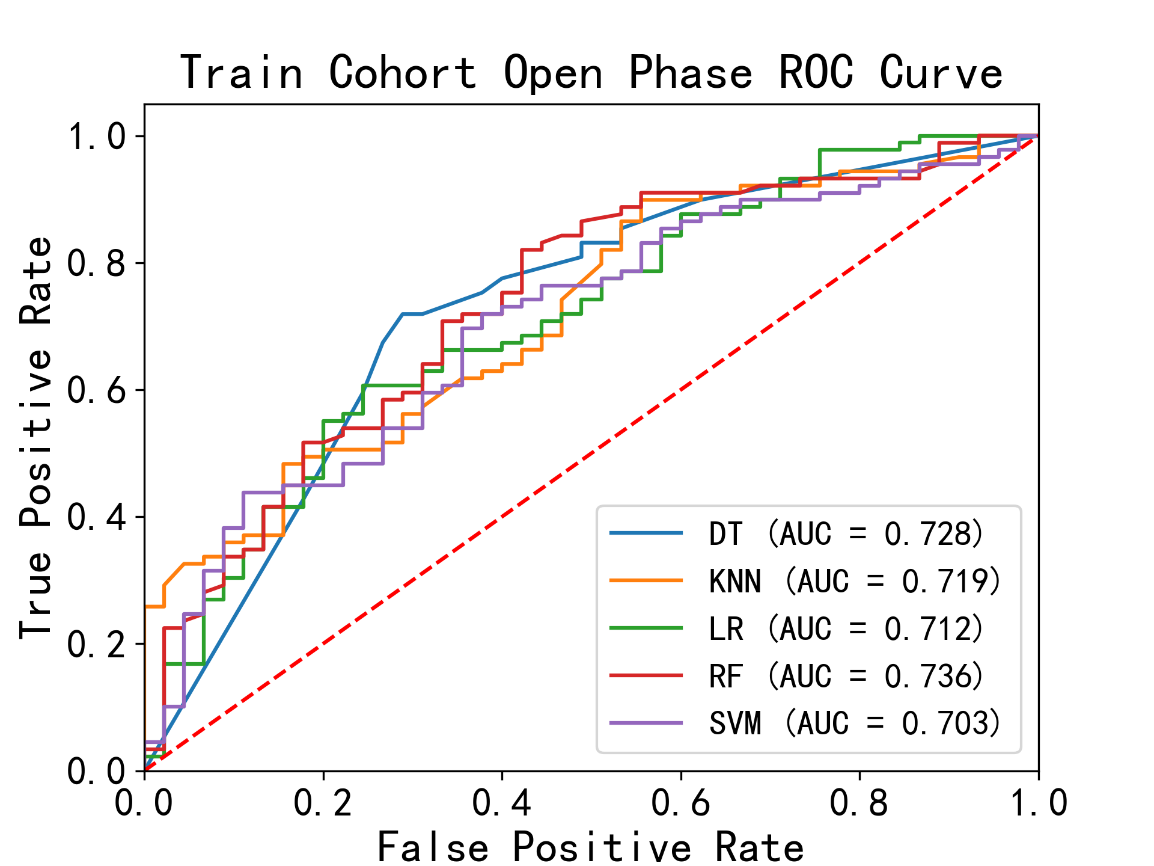


Figure 5A


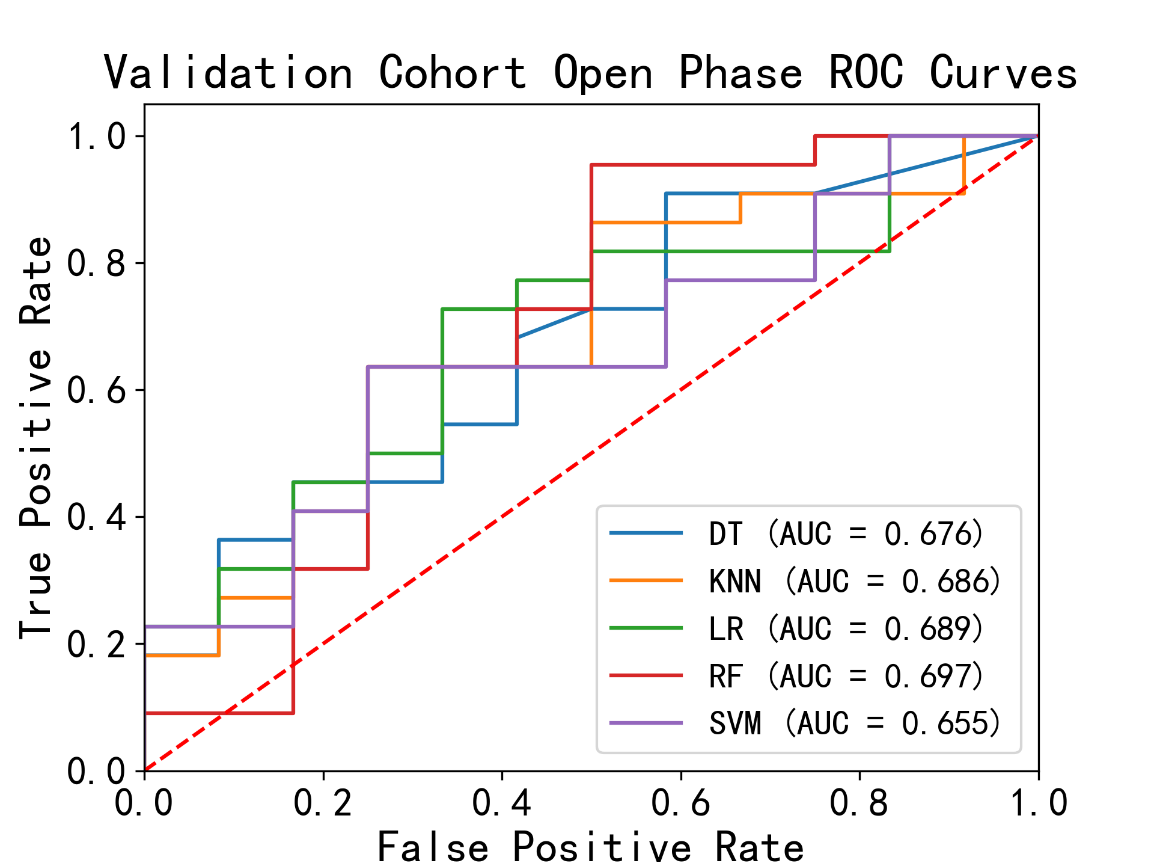


Figure 5B

**Supplementary　Figure 5.**Diagnostic performance of the five models for classifying anterior disc displacement with reduction (ADDwR) and without reduction (ADDwoR) using the open phase in the training (A) and validation (B) cohorts. DT: decision tree; KNN: K-nearest neighbors; LR: logistic regression; RF: random forest; SVM: support vector machine; AUC: area under the curve.


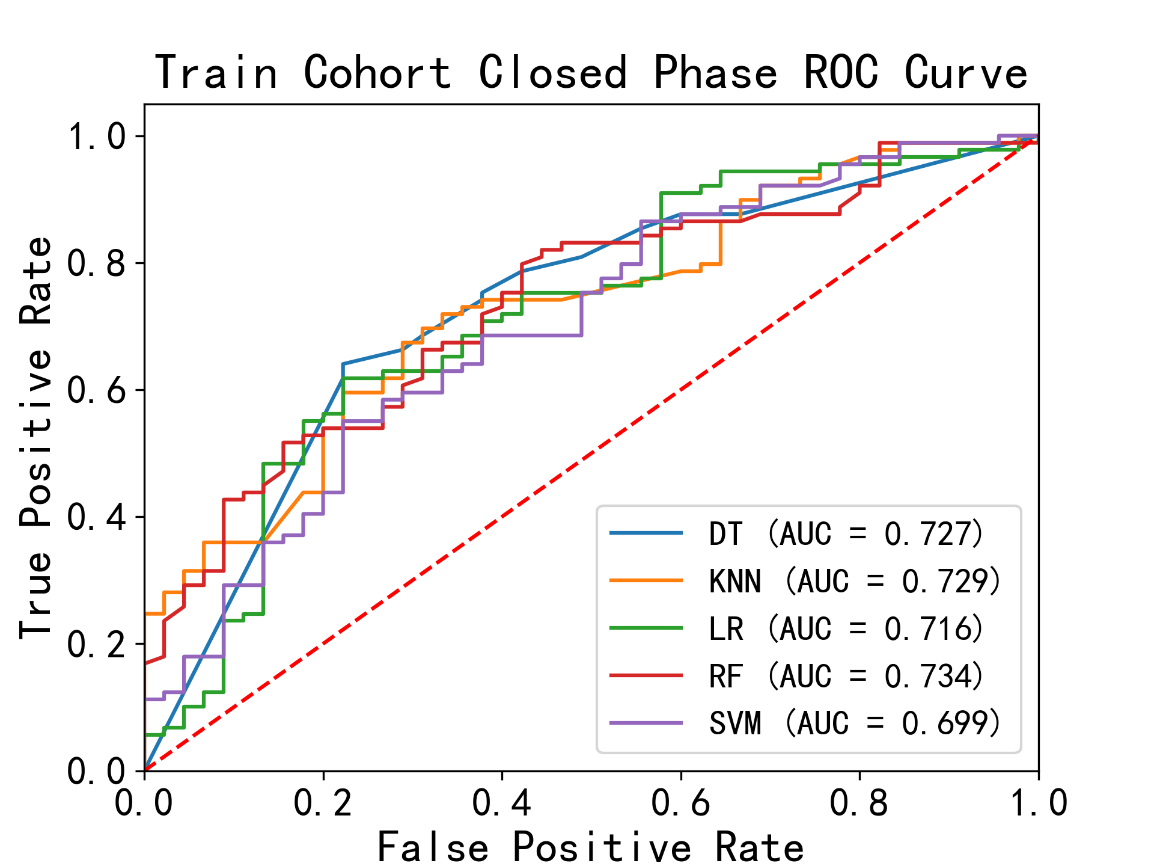


Figure 6A


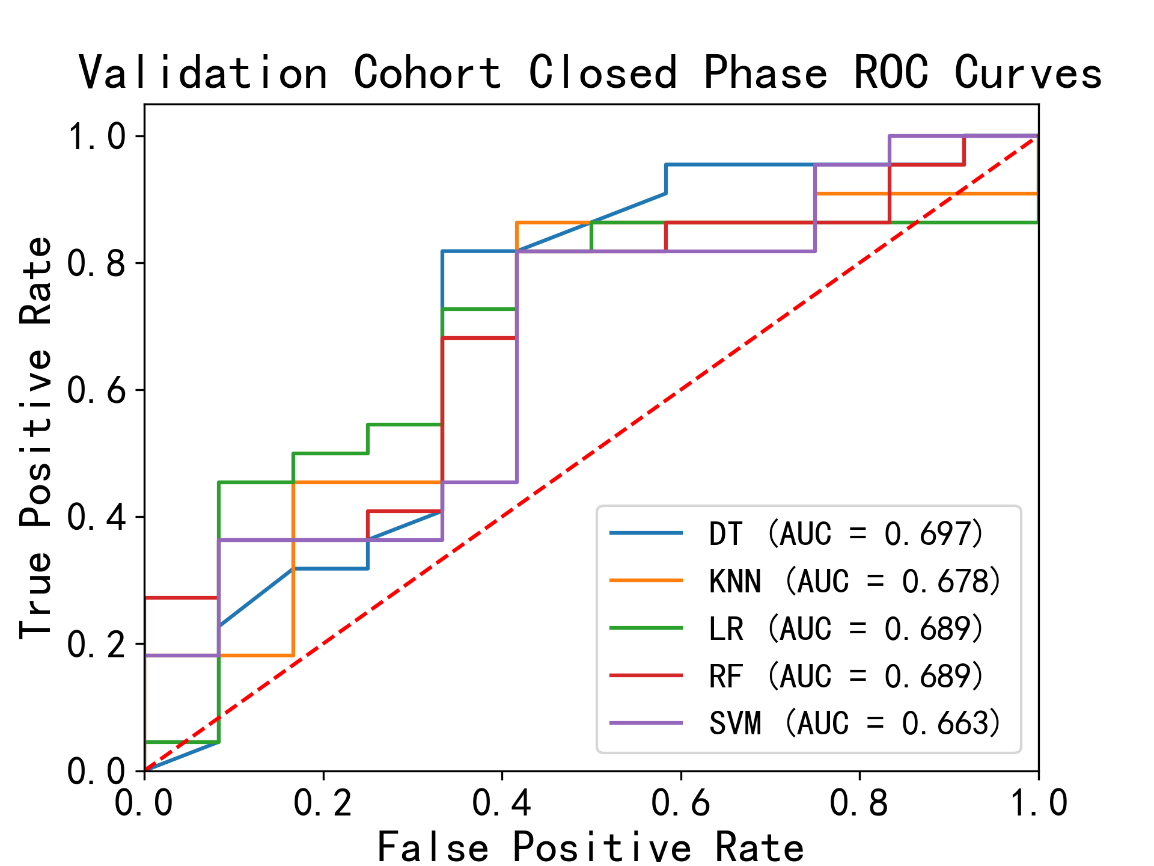


Figure 6B

**Supplementary　Figure 6.**Diagnostic performance of the five models for classifying anterior disc displacement with reduction (ADDwR) and without reduction (ADDwoR) using the close phase in the training (A) and validation (B) cohorts. DT: decision tree; KNN: K-nearest neighbors; LR: logistic regression; RF: random forest; SVM: support vector machine; AUC: area under the curve.
